# Investigation of Non‐*Saccharomyces* Yeasts for Developing Unique Flavor Profiles in Nonalcoholic Mulberry Fermented Beverage

**DOI:** 10.1155/ijfo/5596446

**Published:** 2025-09-19

**Authors:** Chommanat Kerdkhong, Rattiya Padungpol, Chalinee Khongsud, Surak Jamjumrus, Sasiwimol Chansuthep, Weerasak Songoen, Ponsiri Liangsakul, Ulaiwan Withayagiat, Siriphan Sukkhaeng

**Affiliations:** ^1^ Central Laboratory and Greenhouse Complex, Research and Academic Service Center, Faculty of Agriculture at Kamphaeng Saen, Kasetsart University, Kamphaeng Saen Campus, Nakhon Pathom, Thailand, ku.ac.th; ^2^ Department of Agronomy, Faculty of Agriculture at Kamphaeng Saen, Kasetsart University, Kamphaeng Saen Campus, Nakhon Pathom, Thailand, ku.ac.th; ^3^ Department of Biotechnology, Faculty of Agro-Industry, Kasetsart University, Bangkok, Thailand, ku.ac.th

**Keywords:** aroma, *Hanseniaspora*, isoamyl acetate, nonalcoholic wine, non-*Saccharomyce*s

## Abstract

This study presents an innovative exploration of non‐*Saccharomyces* yeast strains isolated from fragrant flowers for use in mulberry juice fermentation, aiming to develop nonalcohol fermented beverages with distinctive and complex aroma profiles. Seventy yeast isolates were screened for sugar utilization efficiency and ethanol production. Thirty‐six strains that produced more than 20‐fold less ethanol than *Saccharomyces cerevisiae* were selected for volatile compound analysis using headspace solid‐phase microextraction gas chromatography–mass spectrometry (HS‐SPME‐GC‐MS). Among these, nine isolates—identified as *Hanseniaspora thailandica*, *Pichia kudriavzevii*, and *Pichia pijperi*—produced a wide range of esters and higher alcohols associated with desirable wine aromas. Notably, *H. thailandica* strain S64‐2 demonstrated the highest aromatic potential, producing elevated levels of isoamyl acetate, phenethyl alcohol, linalool, ethyl acetate, and methyl salicylate—compounds associated with floral and fruity notes. Although *H. thailandica* S64‐2 exhibited ethanol productivity comparable to *S. cerevisiae* under high‐sugar conditions, ethanol concentration was successfully limited to below 0.5% v/v (nonalcoholic beverage standard) through fourfold dilution of the juice and omission of exogenous sugar. A key novelty of this work is to report the fermentation potential of *H. thailandica* S64‐2 in mulberry juice and to provide detailed insights into its volatile profile. Furthermore, the study introduces a new strategy for leveraging indigenous floral yeasts with aroma‐enhancing capabilities to develop nonalcoholic fermented beverages with enriched sensory quality. These findings align with emerging consumer demands and offer a sustainable alternative to traditional wine fermentation practices.

## 1. Introduction

The global market share for nonalcoholic wine has steadily increased in recent years, with expectations for continued growth. Alcohol has become a critical subject of debate from both health and social perspectives, prompting a shift toward nonalcoholic beverages. The maximum allowable alcohol content for nonalcoholic wine is 0.5% by volume [1]. Technological approaches to reducing alcohol in wine are categorized into prefermentation and postfermentation techniques [2]. Postfermentation methods include ethanol removal through vacuum distillation, membrane processes, and evaporation, which require substantial investment. Alternatively, prefermentation strategies, which are more cost‐effective, focus on reducing sugar content through juice dilution or by employing specialized yeast strains with low ethanol production capabilities [2, 3].

Recent studies have increasingly focused on the use of non‐*Saccharomyces* yeast species as an alternative to *Saccharomyces cerevisiae* in low‐alcohol or alcohol‐free winemaking. These yeasts, either used alone or in sequential or coinoculation with *S. cerevisiae*, have demonstrated potential to limit ethanol yield while enhancing aroma complexity [4]. For example, *Metschnikowia pulcherrima* has been shown to reduce ethanol production in sequential fermentations [5], while *Hanseniaspora uvarum*, *Zygosaccharomyces sapae*, and related species were able to produce wines with reduced ethanol in single inoculation fermentations [6]. Similarly, *Hanseniaspora opuntiae* and *H. uvarum* were effective in reducing ethanol levels in medium‐scale fermentations [7]. In addition to their low ethanol output, many non‐*Saccharomyces* strains enhance the formation of desirable volatile aroma compounds, such as esters, higher alcohols, terpenes, aldehydes, and ketones, which contribute to the overall sensory appeal of wine [8–11]. For instance, phenethyl alcohol provides rose‐like aromas, isoamyl alcohol adds whiskey‐like notes, and esters contribute fruity and floral characteristics [12]. A wide range of non‐*Saccharomyces* yeasts—including *Torulaspora delbrueckii*, *Hanseniaspora vineae*, *Starmerella bacillaris*, *Pichia kudriavzevii*, *Pichia anomalus*, and *Lachancea thermotolerans*—have been reported to improve wine’s sensory complexity while offering moderate ethanol reduction [13]. However, most studies to date have achieved only a partial reduction in ethanol content (typically 1%–2%), falling short of the < 0.5% v/v requirement for nonalcoholic classification [5, 7]. Moreover, many studies focus primarily on grape‐based matrices, with limited exploration of other fruit substrates.

The fermentation of nongrape fruits, such as citrus, raspberry, and cherry, has gained attention in recent years to meet consumer demands for diverse flavor profiles and functional benefits [14]. Among them, mulberry (*Morus alba* L.) stands out as a promising substrate due to its high content of polyphenols and bioactive compounds with antioxidant and health‐promoting properties [15]. With the rising interest in functional and nonalcoholic beverages, mulberry wine presents significant potential for innovation. However, to date, there have been no comprehensive studies on the application of non‐*Saccharomyces* yeasts in producing nonalcoholic mulberry‐based fermented beverages with defined aroma profiles. Therefore, significant research gaps remain, including the limited screening of non‐*Saccharomyces* yeast strains from natural or diverse ecological sources such as flowers and fruits, the lack of systematic studies that integrate low ethanol production with aroma enhancement in nongrape substrates, and the minimal efforts to achieve the stringent ethanol threshold of < 0.5% v/v while maintaining yeast viability and the synthesis of aromatic compounds.

In this study, we aimed to address some of these gaps by evaluating the fermentation capacity of non‐*Saccharomyces* yeast strains isolated from fragrant flowers—alongside *S. cerevisiae*—in pasteurized mulberry juice. We assessed both ethanol production and the synthesis of volatile aroma compounds. The final goal was to explore the potential of these indigenous yeasts to develop novel, nonalcoholic fermented beverages with complex and desirable aroma profiles, based on mulberry juice as a functional fruit substrate.

## 2. Materials and Methods

### 2.1. Yeast Isolates and Culture Conditions

Seventy non‐*Saccharomyces* yeast isolates were obtained in 2013 from fragrant flowers in the garden of the Central Laboratory and Greenhouse Complex, Faculty of Agriculture at Kamphaeng Saen, Kasetsart University, Kamphaeng Saen Campus (Supporting Information 1: Table S1). A commercial wine yeast, *S. cerevisiae* (Burgundy), preserved in our laboratory, was included as a reference strain. All isolates were maintained at −80°C in 30% glycerol. The isolates were activated on yeast peptone dextrose (YPD) agar (10 g yeast extract, 20 g peptone, 20 g glucose, and 15 g agar per 1 L distilled water) at 30°C for 48 h.

### 2.2. Preliminary Screening of Non‐*Saccharomyces* Yeasts for Low Ethanol Production

Yeasts were initially screened for glucose consumption and ethanol production following. Each isolate was grown axenically in 5 mL YPD broth (same composition as YPD agar without agar) in 12‐mL tubes. Cultures were incubated at 30°C, 150 rpm for 24 h. Cell density was standardized to 0.5 McFarland, followed by reinoculation of 0.5 mL into fresh 5 mL YPD broth for 72 h under the same conditions. After centrifugation at 8000 rpm for 10 min at 4°C, supernatants were analyzed for glucose using a sucrose/d‐fructose/d‐glucose assay kit (Megazyme, Ireland) and for ethanol using an ethanol assay kit (Megazyme, Ireland). The criteria used for selecting low ethanol‐producing isolates were based on their ability to efficiently utilize glucose while producing ethanol at levels at least 20‐fold lower than *S. cerevisiae*.

### 2.3. Yeast Identification

Based on the results of ethanol production, 36 yeast isolates exhibiting low ethanol yields were selected for molecular identification. DNA from these isolates was extracted following Vingataramin and Frost [16]. The D1/D2 domain of the 26S rDNA was amplified using primers NL1 (5′‐GCATATCAATAAGCGGAGGAAAAG‐3′) and NL4 (5′‐GGTCCGTGTTTCAAGACGG‐3′) [17]. The amplification reactions were performed in a thermal cycler with the following conditions: an initial denaturation at 94°C for 5 min, followed by 45 cycles of denaturation at 94°C for 30 s, annealing at 52°C for 30 s, and extension at 72°C for 30 s, concluding with a final extension at 72°C for 10 min. The PCR products were purified using the GF‐1 AmbiClean Kit (Vivantis, United States) and sequenced by ATGC Co. Ltd. (Thailand). The sequencing results were analyzed by comparing them with those of corresponding type strains using the BLAST service of the National Center for Biotechnology Information (NCBI, http://www.ncbi.nlm.nih.gov/Blast.cgi). Phylogenetic analysis was conducted by aligning the DNA sequences of various non‐*Saccharomyces* species using the general time‐reversible (GTR) model and the maximum‐likelihood analyses performed with MEGA 11 [18]. All positions containing gaps were eliminated. Bootstrap analyses were performed using 1000 random resamplings [19].

### 2.4. Screening of Aroma‐Producing Strain

#### 2.4.1. Lab‐Scale Fermentation

Ripe mulberries (*Morus alba* L.) were used in this study. Due to the high viscosity of undiluted mulberry juice, the fruit was manually crushed and diluted with water (1:4 w/v) to obtain a consistency similar to that of ready‐to‐drink fruit juice. Juice was then filtered through double‐layer cheesecloth to remove fibrous materials, yielding 3.53 g/L glucose and 1.6°Brix. Since glucose in the juice serves as a substrate for ethanol production, its concentration was adjusted to match that used in the initial screening experiments to allow for comparative assessment of ethanol production by the yeast strains. The glucose concentration was adjusted to 20 g/L using d‐glucose and pH to 4.0 using citric acid or NaHCO_3_. Ten‐milliliter aliquots were pasteurized at 85°C for 10 min, cooled, and inoculated with a single colony of similar size from each yeast isolate grown on YM agar (3 g malt extract, 3 g yeast extract, 5 g peptone, 10 g glucose, and 15 g agar per 1 L distilled water). Fermentations were conducted at 25°C for 3 days. Supernatants were centrifuged (8000 rpm, 10 min, 4°C) and analyzed for glucose and fructose using a sucrose/d‐fructose/d‐glucose assay kit (Megazyme, Ireland) and for ethanol using an ethanol assay kit (Megazyme, Ireland). Volatile aroma components were analyzed using headspace solid‐phase microextraction coupled with gas chromatography–mass spectrometry (HS‐SPME‐GC‐MS).

#### 2.4.2. GC‐MS Analysis of Aroma Compounds

Volatiles were extracted from pooled biological triplicates using HS‐SPME with a DVB/CAR/PDMS fiber (50/30 *μ*m, Supelco Ltd., United States). GC‐MS analysis was performed on a single pooled sample without technical replication. As such, the reported data reflect average profiles without statistical evaluation. The pooled sample (5 mL) was transferred into a 20‐mL SPME sample vial. To counter matrix effects and enhance sensitivity, 1.0 g of NaCl was added. Five microliters of 2‐octanol (15 ppm) as an internal standard (final concentration 0.015 ppm) was added to facilitate semiquantitative analysis. The sealed samples were equilibrated in the autosampler at 50°C for 10 min and extracted for 40 min. GC‐MS was performed on a TRACE 1310 (Thermo Scientific, United States) with a TG‐WaxMS fused silica capillary column (30 m × 0.25 mm × 0.25 *μ*m). The sample was then introduced to the gas chromatograph at 230°C for 4 min. Injections were performed at 230°C in splitless mode using a TriPlus RSH Autosampler. The oven program: 40°C for 4 min, ramped to 220°C at 6°C/min, held at 220°C for 6 min (total 40 min). Helium was the carrier gas (1.0 mL/min). Mass spectra were collected at 70 eV (35–500 m/z). Compounds with ≥ 70% similarity in the NIST 17 library were reported. For quantification, 2‐octanol served as the internal standard. Semiquantification was based on the internal standard ratio.

### 2.5. Fermentation Comparison: *Hanseniaspora thailandica* S64‐2 and *S. cerevisiae*

Based on the aroma evaluation results, *H. thailandica* S64‐2 was selected as a representative aroma‐producing strain. To evaluate its fermentation characteristics, a comparative fermentation experiment was conducted with *S. cerevisiae*. The performance of *H. thailandica* S64‐2, selected for unique aroma production, was compared with *S. cerevisiae* in terms of volatile profile, sugar utilization, ethanol production, and cell viability. Mulberry juice with 20 g/L glucose was fermented as described in Section 2.4.1. Viable yeast counts were determined by plate counting (colony‐forming units per milliliter). Ethanol content was quantified by GC‐FID, while glucose and fructose concentrations were measured using HPLC‐ELSD. Aroma compounds were analyzed using HS‐SPME‐GC‐MS with biological triplicates.

### 2.6. Sugar Utilization by *H. thailandica* S64‐2 and *S. cerevisiae*

This experiment was designed to compare the effects of different sugar types—glucose and sucrose, the latter commonly used in traditional winemaking practices—on sugar utilization and ethanol production. The initial glucose concentration was reduced from that used in previous experiments to evaluate whether ethanol production could be lowered below the 0.5% v/v threshold while still supporting yeast growth. In parallel, sucrose was assessed as an alternative carbon source. A control group without any added sugar was included to determine whether the native sugars present in mulberry juice alone were sufficient to support yeast growth and ethanol production. Mulberry juice (1:4 dilution) was supplemented with glucose or sucrose to the final concentrations at 10 and 20 g/L. A control (no sugar added) was included. Natural sugar content of the juice (glucose, fructose, and sucrose) was analyzed prior to sugar supplementation. Single colonies of similar size of *H. thailandica* S64‐2 and *S. cerevisiae* were inoculated into 10 mL of each treatment and fermented at 25°C for 3 days. Supernatants were analyzed for glucose, fructose, and sucrose using HPLC‐ELSD and for ethanol using GC‐FID.

### 2.7. Statistical Analysis

All experiments were performed in triplicate unless stated otherwise. For the screening of aroma‐producing strain, biological triplicates were pooled before HS‐SPME‐GC‐MS analysis, and data are presented as average values without statistical testing. For the assessment of volatile profiles and odor activity values (OAVs), heatmaps and hierarchical cluster analysis (HCA) were generated using the pheatmap package in R. For comparative analysis of aroma compounds and fermentation parameters, one‐way ANOVA with Duncan’s multiple range test (*p* < 0.05) was conducted using SPSS.

## 3. Results and Discussion

### 3.1. Results of Preliminary Screening for Low Ethanol‐Producing Non‐*Saccharomyces* Yeasts

**Figure 1:**
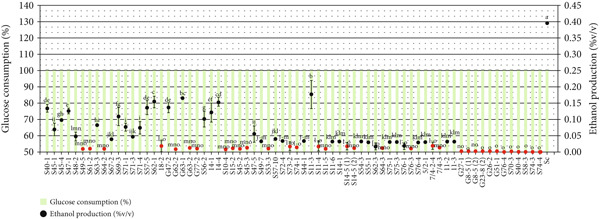
Glucose consumption (%) shown as bar graphs and ethanol production (% v/v) shown as circles of 70 non‐*Saccharomyces* yeast isolates and *Saccharomyces cerevisiae* (Sc; control yeast) after 3 days of fermentation in YPD medium. Identical letters above the circles indicate no significant differences, as determined by Duncan’s multiple range test at *p* < 0.05. Non‐*Saccharomyces* yeasts selected for the next step (ethanol production ≤ 20× that of Sc; ≤ 0.02% v/v) are highlighted in red.

An initial screening was conducted using 70 non‐*Saccharomyces* isolates in YPD broth, a condition characterized by low‐sugar concentration conducive to fermentation, to assess their ability to consume sugars while producing minimal ethanol. This rapid screening technique was previously employed, who identified 14 non‐*Saccharomyces* strains demonstrating a 10% reduction in ethanol production compared to the control strain Sc23. In our study, the 70 non‐*Saccharomyces* yeast isolates were cultured in 5 mL of YPD broth, incubated at 30°C, and shaken at 150 rpm for 3 days. *S. cerevisiae* was used as a control. Under these conditions, *S. cerevisiae* consumed all available glucose and produced 0.39% v/v ethanol, which was lower than the theoretical yield reported by Tae‐Hee et al. [20] (Figure 1). This reduction may be attributed to the low‐sugar content and aerobic fermentation conditions induced by shaking, leading the yeast to prioritize growth over ethanol production [21]. The yeast strains were selected based on their ability to consume a high amount of sugar while producing low levels of ethanol. The results indicated that all tested isolates achieved over 95% efficiency in glucose utilization and produced ethanol in the range of 0.00%–0.18% v/v (Figure 1).

The selection criteria for lower ethanol‐producing yeast isolates required a reduction in ethanol production by 20‐fold compared to *S. cerevisiae*. Consequently, 36 non‐*Saccharomyces* isolates were chosen based on their high glucose consumption and significantly lower ethanol yields, which were less than 0.02% v/v. This selection included strains S49‐5, S61‐2, S64‐2, 18‐2, G62‐2, G63‐2, G77‐2, S10‐4, S15‐2, S45‐2, S45‐3, S53‐3, S73‐2, S74‐1, S11‐4, S11‐5, S14‐5(1), S14‐5(2), S62‐3, S64‐3, S76‐1, S76‐3, 7/4‐2‐4, 7/4‐3, G27‐1, G8‐5(1), G8‐5(2), G23‐8(2), G26‐2, G51‐1, G76‐4, S70‐3, S40‐4, S58‐3, S74‐3, and S74‐4 (Figure 1).

These findings demonstrate that several non‐*Saccharomyces* isolates can efficiently utilize glucose while producing significantly lower ethanol levels compared to *S. cerevisiae*, suggesting a predominantly respiratory metabolism under the tested conditions. The wide range of ethanol production among isolates highlights strain‐dependent variability in metabolic behavior. Selecting isolates with over 20‐fold reduction in ethanol production ensures a robust starting point for further investigation into their potential in low‐alcohol or nonalcoholic beverage fermentation. These selected strains were particularly notable for their low ethanol output and will be further identified at the species level and examined for their aroma‐producing potential in subsequent experiments.

### 3.2. Identification of Non‐*Saccharomyces*

**Figure 2:**
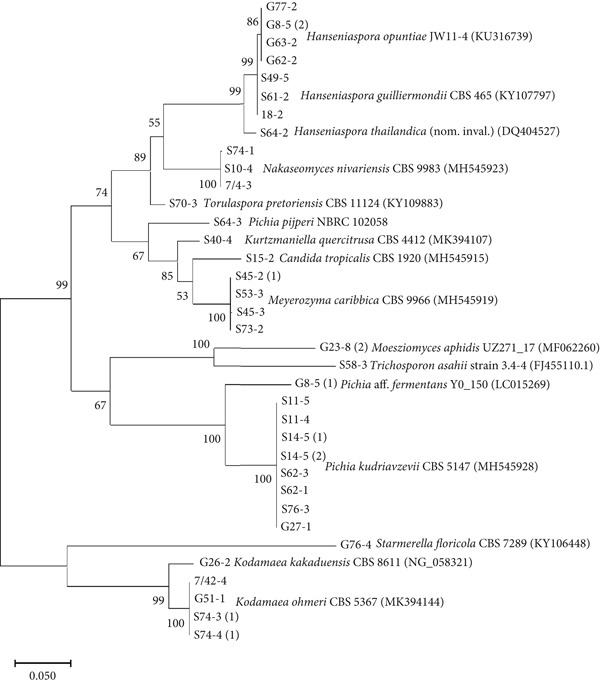
Phylogenetic tree of 36 non‐*Saccharomyces* yeast isolates obtained from fragrant flowers based on the sequence analysis of the 26S rRNA D1/D2 region using the maximum‐likelihood method. The scale bar shows 0.05. Bootstrap support values were estimated based on 1000 replicates and are shown above the branches (> 50%).

Yeast species were identified by sequencing the D1/D2 region of the 26S rDNA. All 36 non‐*Saccharomyces* yeast isolates were identified as described species, belonging to 16 species across 12 genera: *Hanseniaspora guilliermondii*, *H. thailandica*, *H. opuntiae*, *Nakaseomyces nivariensis*, *Candida tropicalis*, *Meyerozyma caribbica*, *P. kudriavzevii*, *Pichia pijperi* (syn. *Wickerhamomyces pijperi*), *Kodamaea ohmeri*, *Pichia aff. fermentans*, *Kodamaea kakaduensis*, *Starmerella floricola*, *Torulaspora pretoriensis*, *Kurtzmaniella quercitrusa*, *Moesziomyces aphidis*, and *Trichosporon asahii* (Supporting Information 2: Table S2). Most isolates belonged to the phylum Ascomycota, while a few were members of Basidiomycota. The phylogenetic tree constructed from D1/D2 sequences confirmed the identity of each isolate (Figure 2). Genera *Hanseniaspora* and *Pichia* were the most frequently represented, with *P. kudriavzevii* as the most predominant species. Several species identified in this study, including *H. guilliermondii*, *H. opuntiae*, *H. thailandica*, *P. kudriavzevii*, and *T. asahii*, have been previously associated with winemaking [22–26].

### 3.3. Screening Results of Aroma‐Producing Strains Using SPME‐GC‐MS

In light of the health benefits associated with mulberry wine and its potential as a nonalcoholic beverage, we further screened non‐*Saccharomyces* strains for their suitability and aromatic profiles in fermented mulberry juice. Thirty‐six strains from the initial selection were used to ferment pasteurized mulberry juice over 3 days. The control group consisted of 1:4 diluted mulberry juice, which served as the fermentation base for both *S. cerevisiae* and the non‐*Saccharomyces* strains. Volatile profiles were analyzed using solid‐phase microextraction coupled with gas chromatography–mass spectrometry (SPME‐GC‐MS). The concentrations (micrograms per liter) of each volatile compound across the 37 wine samples and mulberry juice are detailed in Supporting Information 3: Table S3. A total of 71 volatile compounds were identified and categorized into eight chemical classes: alcohols (15), aldehydes (3), ketones (9), acids (14), esters (10), hydrocarbons (7), phenols (9), and miscellaneous compounds (4). The total concentrations of these volatiles were compared using a heatmap and dendrogram (Figure 3a). Based on ester and alcohol production, the strains were divided into two groups: 27 strains, including *S. cerevisiae* and the unfermented mulberry juice, produced lower levels of these compounds, while 9 strains exhibited relatively higher production. These included *H. thailandica* S64‐2, *P. kudriavzevii* strains (S76‐1, S76‐3, S11‐4, S62‐3, S11‐5, S14‐5[2], and G27‐1), and *P. pijperi* S64‐3. Notably, *H. thailandica* S64‐2 was distinguished by its high production of esters, which are key contributors to fruity aroma. The fruity character of red wine has been attributed to perceptual interactions among various volatile compounds, particularly ethyl esters and acetates, whereas higher alcohols, although contributing to aromatic complexity, may in some cases mask desirable flavors [27]. According to Torrens et al. [28], yeast strains that produce high levels of acetates, esters, and phenylethanol can enhance wine’s sensory attributes. Yeasts from the genera identified in this study have also been associated with improved physicochemical and sensory qualities in wine [13].

Figure 3(a) Overview of the total volatile compound content (micrograms per liter) across different chemical groups for 36 yeast strains. (b) Overview of selected volatile compounds expressed as odor activity values (OAVs) for nine yeast strains with high total volatile production. Red: relatively high production; blue: relatively low production.

**Figure:**
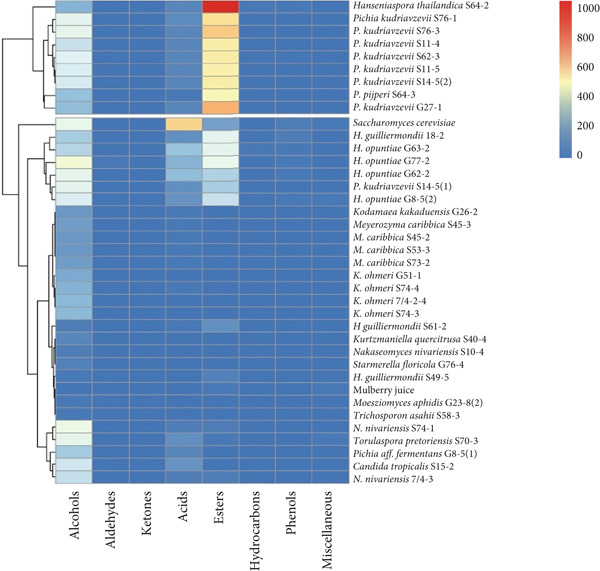
(a)

**Figure:**
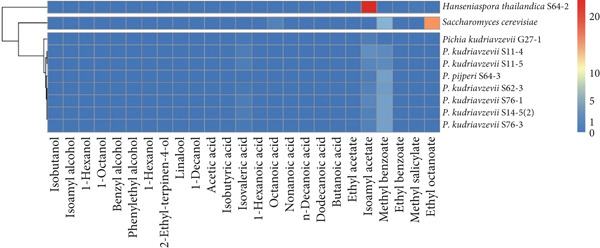
(b)

OAVs of important volatiles from esters, acids, and alcohols were analyzed for the nine strains that produced higher levels of these compounds and compared with *S. cerevisiae* (Supporting Information 4: Table S4). According to Li et al. [9], OAVs quantify the likelihood that a volatile compound will be perceived as an odorant by comparing its concentration to its odor threshold. While compounds with OAVs greater than 1 are considered to have a direct impact on aroma, those with values between 0.1 and 1 may still influence overall perception through synergistic or masking effects. The OAVs for each major volatile compound were also visualized and compared using a heatmap and dendrogram (Figure 3b). *H. thailandica* S64‐2 was notable for producing a substantial quantity of isoamyl acetate, the primary ester formed during fermentation, in contrast to *S. cerevisiae*, which primarily produces ethyl octanoate and methyl benzoate. To further quantify the relative contribution of each compound, relative OAVs (rOAVs, scaled from 0 to 100) were calculated by dividing each compound’s OAV by the highest OAV in the dataset (Supporting Information 4: Table S4). Although Supporting Information 4: Table S4 shows that other strains produced a wider range of volatiles than *H. thailandica* S64‐2, this strain was selected for further study due to its ability to elevate key aroma‐active compounds, as revealed by the OAV‐based screening results. The focus of this work was not overall volatile diversity, but the generation of high‐impact odorants. *H. thailandica* S64‐2 produced an intense and targeted aroma profile, particularly through isoamyl acetate, known for its strong fruity notes [29, 30]. Furthermore, the selection of this strain for further study was also supported by preliminary aroma impressions observed during fermentation.

However, a key limitation of this study is the use of single‐colony inoculation. While efforts were made to select colonies of similar size, slight variations in initial cell numbers may have affected fermentation dynamics and metabolite output. Future studies should include inoculum standardization to enhance reproducibility and data accuracy.

### 3.4. Comparison of Major Aroma Compounds and Fermentation Behavior Between *H. thailandica* S64‐2 and *S. cerevisiae*

The aromatic profile of *H. thailandica* S64‐2 differed from that obtained with *S. cerevisiae* (Table 1), particularly due to the production of high alcohols (isoamyl alcohol, 1‐octanol, benzyl alcohol, and phenylethyl alcohol), terpenes (linalool), acids (acetic acid, nonanoic acid, benzoic acid, and dodecanoic acid), esters (ethyl acetate, isoamyl acetate, benzyl acetate, and methyl salicylate), and phenols (carvacrol). *Hanseniaspora* species are generally known as high producers of acetate esters due to their esterase activity, which can modulate wine flavor by increasing levels of certain ethyl and acetate esters [37, 38]. Although phenylethyl acetate was not detected in this study, previous reports have linked its production to *Hanseniaspora* strains. Moreover, the higher *β*‐glucosidase activity typically observed in *Hanseniaspora* compared to *S. cerevisiae* may explain the increased levels of terpenes, such as linalool, which contribute to varietal aroma [25, 38]. The OAVs are typically used to provide a rough evaluation of the contribution of each aroma compound to the overall aroma. Generally, volatile compounds with OAVs greater than 1 are considered important aroma compounds [9]. In this study, isoamyl acetate is a key aroma compound in wine produced through fermentation with *H. thailandica* S64‐2. Compounds with OAVs between 0.1 and 1 may influence the aroma through interactions with other aroma components, such as phenylethyl alcohol, linalool, ethyl acetate, and methyl salicylate. Among these aroma compounds, isoamyl acetate imparts a banana aroma, phenylethyl alcohol provides honey and rose notes, linalool and ethyl acetate impart floral and fruity aromas, and methyl salicylate has minty and balsamic odors [12, 31]. These desirable compounds contribute pleasant floral or fruity aromas to wines. In contrast, the main aroma in wine produced through fermentation with *S. cerevisiae* was ethyl octanoate, while the secondary component was octanoic acid. This ethyl ester imparts a fruity aroma, while octanoic acid, a fatty acid, provides an unpleasant aroma reminiscent of rancid or cheese‐like notes [12]. In addition, *H. thailandica* was used to enhance the flavor in tangerine wine and plum wine through fermentation under conditions with an initial sugar content of approximately 130–250 g/L, which elevated the levels of isoamyl acetate and other volatiles, surpassing those produced by *S. cerevisiae* [24, 39].

**Table 1:** Concentrations (micrograms per liter) of volatile aroma compounds in diluted mulberry juice (MJ) and wines fermented with *Hanseniaspora thailandica* S64‐2 (Ht) were compared with those fermented using *Saccharomyces cerevisiae* (Sc).

**Rt**	**Compounds**	**MJ**	**Ht**	**Sc**	**Odor threshold (*μ*g/L)**
8.788	Isoamyl alcohol ^∗∗∗^	1.49 ± 0.18^c^	112.13 ± 11.84^a^	21.74 ± 1.57^b^	30,000^I^
12.342	1‐Hexanol ^∗∗∗^	nd^b^	nd^b^	0.06 ± 0.01^a^	500^II^
13.315	2‐Hexanol, 2‐methyl‐ ^∗∗∗^	nd^b^	nd^b^	0.43 ± 0.01^a^	N/A
15.424	1‐Hexanol, 2‐ethyl‐ ^∗∗∗^	10.57 ± 0.83^a^	11.02 ± 1.97^a^	0.69 ± 0.08^b^	270^II^
15.431	2‐Propyl‐1‐pentanol ^∗∗∗^	2.36 ± 0.15^a^	nd^b^	0.09 ± 0.01^b^	N/A
16.846	1‐Octanol ^∗∗∗^	0.82 ± 0.03^b^	3.05 ± 0.48^a^	0.32 ± 0.02^c^	110^II^
18.902	1‐Undecanol ^∗∗∗^	nd^b^	nd^b^	0.75 ± 0.00^a^	N/A
20.840	1‐Decanol ^∗∗∗^	nd^b^	nd^b^	0.44 ± 0.04^a^	400^III^
22.929	Benzyl alcohol ^∗∗∗^	nd^b^	18.06 ± 1.20^a^	nd^b^	620^IV^
23.516	Phenylethyl alcohol ^∗∗∗^	nd^b^	117.93 ± 13.48^a^	9.99 ± 0.46^b^	110^II^
	∑ Alcohols ^∗∗∗^	15.24 ± 0.97^c^	255.16 ± 11.99^a^	34.09 ± 1.46^b^	—
7.707	Linalool ^∗∗^	4.38 ± 0.60^b^	8.93 ± 2.74^a^	0.48 ± 0.04^c^	15^I^
	∑ Terpenes ^∗∗^	4.38 ± 0.60^b^	8.93 ± 2.74^a^	0.48 ± 0.04^c^	—
13.117	Nonanal ^∗∗∗^	0.54 ± 0.10^a^	nd^b^	nd^b^	1^V^
16.085	Benzaldehyde ^∗∗∗^	4.95 ± 0.38^a^	nd^b^	nd^b^	350^VI^
18.516	Benzeneacetaldehyde ^∗∗∗^	1.01 ± 0.18^a^	nd^b^	Nd^b^	N/A
	∑ Aldehydes ^∗∗∗^	6.51 ± 0.52^a^	nd^b^	nd^b^	
14.750	Acetic acid ^∗^	nd^b^	30.01 ± 17.40^a^	0.67 ± 0.46^b^	200,000^VII^
17.181	Propanoic acid, 2‐methyl‐ ^∗∗∗^	nd^b^	nd^b^	0.12 ± 0.03^a^	2300^VII^
22.433	Hexanoic acid ^∗∗∗^	nd^b^	nd^b^	4.72 ± 0.52^a^	420^I^
24.217	Hexanoic acid, 2‐ethyl‐ ^∗∗∗^	0.92 ± 0.20^a^	nd^b^	nd^b^	N/A
26.048	Octanoic acid ^∗∗∗^	1.37 ± 0.16^c^	36.50 ± 1.03^b^	70.14 ± 6.25^a^	500^I^
27.732	Nonanoic acid ^∗^	0.99 ± 0.25^b^	4.00 ± 1.78^a^	0.42 ± 0.09^b^	3000^V^
29.338	n‐Decanoic acid ^∗∗∗^	nd^b^	48.90 ± 2.04^a^	47.97 ± 6.62^a^	1000^VII^
30.244	Undecylenic acid ^∗∗∗^	nd^b^	nd^b^	2.82 ± 0.38^a^	N/A
31.635	Benzoic acid ^∗^	nd^b^	1.17 ± 0.55^a^	0.10 ± 0.02^b^	N/A
32.360	Dodecanoic acid ^∗∗∗^	nd^c^	6.76 ± 0.57^a^	0.92 ± 0.12^b^	1000^IV^
	∑ Acids ^∗∗∗^	3.27 ± 0.48^b^	125.62 ± 18.27^a^	127.80 ± 13.77^a^	—
2.379	Ethyl acetate ^∗∗∗^	21.92 ± 2.94^b^	807.84 ± 75.89^a^	18.04 ± 1.46^b^	7500^IV^
6.440	Isoamyl acetate ^∗∗∗^	4.29 ± 0.71^b^	1478.06 ± 71.36^a^	2.76 ± 0.24^b^	30^I^
18.469	Ethyl octanoate ^∗∗∗^	nd^b^	nd^b^	18.33 ± 1.87^a^	5^I^
20.223	Benzyl acetate ^∗∗∗^	nd^b^	6.77 ± 1.57^a^	nd^b^	N/A
21.035	Methyl salicylate ^∗^	2.94 ± 0.15^b^	15.57 ± 6.60^a^	0.70 ± 0.20^b^	50^I^
	∑ Esters ^∗∗∗^	29.15 ± 3.67^b^	2308.23 ± 127.14^a^	39.60 ± 2.33^b^	—
28.480	Carvacrol ^∗∗^	0.68 ± 0.06^b^	5.97 ± 2.45^a^	0.11 ± 0.02^b^	N/A
29.969	2,4‐Di‐tert‐butylphenol ^∗^	8.53 ± 4.45^ab^	18.52 ± 9.82^a^	1.26 ± 0.40^b^	200^V^
	∑ Phenols ^∗^	9.22 ± 4.42^b^	24.49 ± 11.49^a^	1.37 ± 0.39^b^	—

*Note:* Values with the same letters in the same row are not significantly different according to Duncan’s multiple range test (95%). Odor threshold obtained from literature: Slaghenaufi et al. [31] (superscript I), Wei et al. [32] (superscript II), Zhang et al. [33] (superscript III), Blanco et al. [34] (superscript IV), Avellone et al. [35] (superscript V), Wang et al. [15] (superscript VI), and Francis and Newton [36] (superscript VII).

Abbreviation: nd, not detected.

^***^Significance level of the one‐way ANOVA ( ^∗^0.05 > *p* > 0.01;  ^∗∗^0.01 > *p* > 0.001;  ^∗∗∗^ *p* < 0.001).

**Figure 4:**
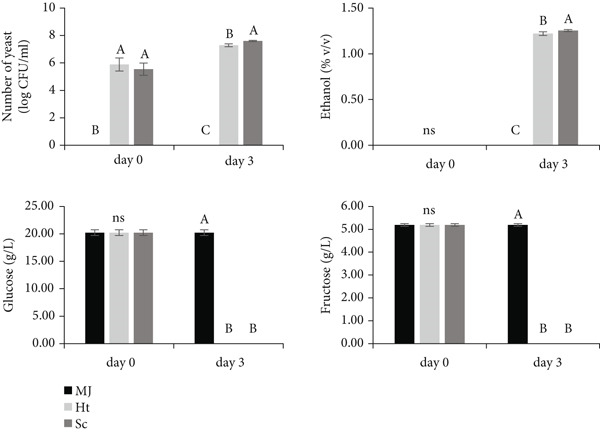
Yeast growth, ethanol production, residual glucose, and fructose contents in diluted mulberry juice (MJ) and wines fermented with *Hanseniaspora thailandica* S64‐2 (Ht) were compared with those fermented using *Saccharomyces cerevisiae* (Sc). Note: Values with the same letters are not significantly different according to Duncan’s multiple range test (95%). ns: no significant difference.

The fermentation efficiency of *H. thailandica* S64‐2 was comparable to that of *S. cerevisiae*. Both strains fully consumed the initial sugars (20 g/L glucose and 5 g/L fructose) within 3 days. However, *H. thailandica* S64‐2 exhibited slightly lower growth and ethanol production, yielding 1.22% v/v ethanol compared to 1.25% v/v by *S. cerevisiae* (Figure 4). This aligns with previous reports indicating that *Hanseniaspora* species require over 19 g/L of sugar to produce 1% v/v ethanol and generally have lower ethanol yields than *S. cerevisiae* [24, 40]. Although *H. thailandica* S64‐2 produced over 20‐fold less ethanol than *S. cerevisiae* in the initial screening using synthetic medium (20 g/L glucose), this study in mulberry juice with the same glucose concentration showed comparable ethanol levels. This discrepancy likely results from matrix effects, as juice components may influence yeast metabolism, particularly for non‐*Saccharomyces* strains [32]. The improved ethanol production by *H. thailandica* S64‐2 in real juice suggests that its performance is environment‐dependent, emphasizing the importance of matrix‐specific evaluations when selecting strains for practical applications.

### 3.5. Sugar Utilization Between *H. thailandica* S64‐2 and *S. cerevisiae*

From previous experiments using mulberry juices with an initial glucose concentration of 20 g/L, *H. thailandica* S64‐2 produced ethanol exceeding 0.5% v/v, which is still not the suitable characteristic for nonalcoholic wine. Therefore, we tested appropriate types and amounts of sugar in fermentation to obtain ethanol less than 0.5% v/v. Table 2 shows that *H. thailandica* S64‐2 utilized glucose effectively; both 10 and 20 g/L concentrations were tested, and ethanol production was comparable to that of *S. cerevisiae*. However, *H. thailandica* S64‐2 was unable to utilize sucrose as effectively as *S. cerevisiae*, resulting in residual sucrose and lower ethanol production. This may be attributed to the invertase enzyme responsible for converting sucrose into glucose and fructose. Non‐*Saccharomyces* species have been reported to exhibit lower invertase enzyme activity than *S. cerevisiae* [41]. Although the conditions that caused *H. thailandica* S64‐2 to produce less than 0.5% v/v ethanol (which align with our objectives) included mulberry juice without added sugar, as well as the addition of 1% glucose and 1% or 2% sucrose, not adding sugar is an economical way to reduce fermentation costs.

**Table 2:** Ethanol production (% v/v) and residual sugar content in wines (grams per liter), that is, glucose (Glu), fructose (Fruc), and sucrose (Suc) after 3 days of fermentation of *Hanseniaspora thailandica* S64‐2 (Ht) and *Saccharomyces cerevisiae* (Sc) in mulberry juice (MJ) and mulberry juice supplemented with 10–20 g/L Glu and 10–20 g/L Suc.

**Treatment (sugar added)**	**Yeast**	**Ethanol production (% v/v)** ^∗∗∗^	**Residual sugar content (g/L)**		
			**Glu** ^∗∗∗^	**Fruc** ^∗∗∗^	**Suc** ^∗∗∗^
MJ 1:4 (no sugar added)	No yeast	nd^j^	2.94 ± 0.30^d^	3.25 ± 0.69^c^	nd^d^
	Ht	0.22 ± 0.01^h^	nd^e^	0.30 ± 0.11^e^	nd^d^
	Sc	0.19 ± 0.01^i^	nd^e^	nd^e^	nd^d^

10 g/L Glu	No yeast	nd^j^	9.51 ± 1.04^b^	4.97 ± 0.62^b^	nd^d^
	Ht	0.45 ± 0.00^f^	nd^e^	nd^e^	nd^d^
	Sc	0.48 ± 0.00^e^	nd^e^	nd^e^	nd^d^

20 g/L Glu	No yeast	nd^j^	18.84 ± 1.50^a^	5.59 ± 0.74^b^	nd^d^
	Ht	1.01 ± 0.02^c^	nd^e^	0.76 ± 0.25^de^	nd^d^
	Sc	1.06 ± 0.00^b^	nd^e^	0.77 ± 0.21^de^	nd^d^

10 g/L Suc	No yeast	nd^j^	6.37 ± 0.63^c^	6.87 ± 0.33^a^	5.92 ± 0.15^b^
	Ht	0.34 ± 0.00^g^	nd^e^	nd^e^	4.74 ± 0.06^c^
	Sc	0.63 ± 0.00^d^	nd^e^	0.74 ± 0.22^de^	nd^d^

20 g/L Suc	No yeast	nd^j^	7.31 ± 0.82^c^	7.65 ± 0.55^a^	14.33 ± 0.15^a^
	Ht	0.44 ± 0.01^f^	nd^e^	nd^e^	14.29 ± 0.25^a^
	Sc	1.22 ± 0.00^a^	nd^e^	1.59 ± 0.43^d^	nd^d^

*Note:* Values with the same letters in the same column are not significantly different according to Duncan′s multiple range test (95%).

Abbreviation: nd, not detected.

^***^Significance level of the one‐way ANOVA (*p* < 0.001).

## 4. Conclusion

The growing interest in the health benefits of mulberry consumption has spurred research into developing a nonalcoholic yet highly nutritious beverage with an appealing flavor profile through fermentation. Seventy non‐*Saccharomyces* strains isolated from fragrant flowers in the yeast culture collection were screened for their ability to produce significantly lower levels of ethanol. Thirty‐six non‐*Saccharomyces* isolates were selected based on their high glucose utilization and reduced ethanol production. Nine strains, identified as *H. thailandica*, *P. kudriavzevii*, and *P. pijperi* (*Wickerhamomyces pijperi*), produced relatively high amounts of esters and alcohols. Notably, *H. thailandica* S64‐2 exhibited a unique volatile profile in fermented mulberry juice, predominantly producing isoamyl acetate, which contributed significantly to the aroma under these conditions. The volatile compounds, phenethyl alcohol, linalool, ethyl acetate, and methyl salicylate may enhance floral and sweet characteristics during the fermentation process. This strain holds promise for use in the fermentation of mulberry juice to develop a novel nonalcoholic beverage. However, to achieve an ethanol content of less than 0.5% v/v in the final product, it is necessary to control the initial sugar content by diluting the mulberry juice fourfold, refraining from adding sugar, and allowing the yeast to utilize the natural sugars present in the fruit juice.

## Conflicts of Interest

The authors declare no conflicts of interest.

## Funding

This work was supported by the Kasetsart University Research and Development Institute (KURDI), Kasetsart University, Bangkok, Thailand (RS[KU]4.65).

## Supporting Information

Additional supporting information can be found online in the Supporting Information section.

## Supporting information


**Supporting Information 1** Table S1: Seventy yeast isolates from various ornamental flowers and their codes.

**Supporting Information 2** Table S2: Identification of 36 isolated non‐*Saccharomyces* yeast strains based on the sequences of the D1/D2 domain of the 26S rDNA gene.

**Supporting Information 3** Table S3: Concentrations (micrograms per liter) of volatile aroma compounds in wines fermented with different yeast strains and diluted mulberry juice (MJ).

**Supporting Information 4** Table S4: Relative odor activity values (rOAVs) of volatile aroma compounds in mulberry wines fermented with different yeast strains.

## Data Availability

The data used to support the findings of this study are included within the article. However, any other information required is available from the corresponding author upon request.
