# Supplementary material for: Investigation of Non‐Saccharomyces Yeasts for Developing Unique Flavor Profiles in Nonalcoholic Mulberry Fermented Beverage
Source: Int J Food Sci. 2025 Sep 19;2025:5596446. doi: 10.1155/ijfo/5596446 (PMC12447106; doi:10.1155/ijfo/5596446)
Supplement: Supplementary file 1 — Supporting Information 1 Table S1: Seventy yeast isolates from various ornamental flowers and their codes. [file IJFO-2025-5596446-s001.docx]

**Table S1.** Seventy yeast isolates from various fragrant flowers and their codes.

| No. | Code | Source |
| --- | --- | --- |
| 1 | S40-1 | *Rosa damascena* Mill. |
| 2 | S45-1 | *Desmos chinensis* Lour. |
| 3 | S45-4 | *Desmos chinensis* Lour. |
| 4 | S47-1 | *Cananga odorara* (Lamk.) Hook.f.& Th var. Odorata |
| 5 | S48-2 | *Mitrephora wangii* Hu |
| 6 | S49-5 | *Quisqualis indica* L. |
| 7 | S61-2 | *Ascocentrum minatum* (Lindl.) |
| 8 | S63-3 | *Tabernaemontana divaricata* (l.) R.Br. Ex Roem & Schult |
| 9 | S64-2 | *Vallaris solanacea* (Roth) Kuntze |
| 10 | S67-2 | *Duranta ereecta* Linn. |
| 11 | S69-3 | *Oncoba spinosa* Forsk. |
| 12 | S71-1 | *Mirabilis jalapa* L. |
| 13 | S71-3 | *Mirabilis jalapa* L. |
| 14 | S71-4 | *Mirabilis jalapa* L. |
| 15 | S57-5 | *Alpinia nigra* B.L. Burtt. |
| 16 | S62-1 | *Gardenia jasminoides* |
| 17 | 18-2 | *Glycosmis pentaphylla* (Retz.) DC. |
| 18 | G47-2 | *Cananga odorara* (Lamk.) Hook.f.& Th var. Ododrata |
| 19 | G62-2 | *Gardenia jasminoides* |
| 20 | G63-1 | *Tabernaemontana divaricata* (l.) R.Br. Ex Roem & Schult |
| 21 | G63-2 | *Tabernaemontana divaricata* (l.) R.Br. Ex Roem & Schult |
| 22 | G77-2 | *Carissa macrocarpa* (Ecklon) A.DC. |
| 23 | S56-2 | *Dellenia indica* L. |
| 24 | 14-1 | *Oncoba spinosa* Forsk. |
| 25 | 14-4 | *Oncoba spinosa* Forsk. |
| 26 | S10-4 | *Hymenocallis littoralis* Salisb. |
| 27 | S15-2 | *Pterospermum littorale* Craib var. lttorale |
| 28 | S45-2 | *Desmos chinensis* Lour. |
| 29 | S45-3 | *Desmos chinensis* Lour. |
| 30 | S47-5 | *Cananga odorara* (Lamk.) Hook.f.& Th var. Odorata |
| 31 | S49-7 | *Quisqualis indica* L. |
| 32 | S53-3 | *Gustavia gracillima* Miers |
| 33 | S57-10 | *Alpinia nigra* B.L. Burtt. |
| 34 | S72-4 | *Lawsonia inermis* L. |
| 35 | S73-2 | *Hymenocallis littoralis* Salisb. |
| 36 | S74-1 | *Crinum asiaticum* Linn. |
| 37 | S44-1 | *Vitex negundo* Linn. |
| 38 | S11-3 | *Goniothalamus laoticus* (Finet & Gagnep.) Ban |
| 39 | S11-4 | *Goniothalamus laoticus* (Finet & Gagnep.) Ban |
| 40 | S11-5 | *Goniothalamus laoticus* (Finet & Gagnep.) Ban |
| 41 | S11-6 | *Goniothalamus laoticus* (Finet & Gagnep.) Ban |
| 42 | S14-1 | *Oncoba spinosa* Forsk. |
| 43 | S14-5(1) | *Oncoba spinosa* Forsk. |
| 44 | S14-5(2) | *Oncoba spinosa* Forsk. |
| 45 | S54-3 | *Buddleja paniculata* Wall. |
| 46 | S55-1 | *Dracaena loureiri* Gagnep |
| 47 | S62-3 | *Gardenia jasminoides* |
| 48 | S64-3 | *Vallaris solanacea* (Roth) Kuntze |
| 49 | S75-1 | *Duranta repens* Linn. |
| 50 | S75-3 | *Duranta repens* Linn. |
| 51 | S76-1 | *Barringtonia asiatica* (L.) Kurz |
| 52 | S76-3 | *Barringtonia asiatica* (L.) Kurz |
| 53 | S76-4 | *Barringtonia asiatica* (L.) Kurz |
| 54 | 5/2-1 | *Jasminum adenophyllum* Wall. ex C.B. Clarke |
| 55 | 7/4-2-4 | *Pterospermum littorale* Craib var. lttorale |
| 56 | 7/4-3 | *Pterospermum littorale* Craib var. lttorale |
| 57 | 11-2 | *Goniothalamus laoticus* (Finet & Gagnep.) Ban |
| 58 | 11-3 | *Goniothalamus laoticus* (Finet & Gagnep.) Ban |
| 59 | G27-1 | *Hibiscus rosa-sinensis* L. |
| 60 | G8-5(1) | *Clerodendrum chinensis* (Osbeck) Mabb. |
| 61 | G8-5(2) | *Clerodendrum chinensis* (Osbeck) Mabb. |
| 62 | G23-8(2) | *Jatropha multifida* L. |
| 63 | G26-2 | *Plumeria obtusa* Linn. |
| 64 | G51-1 | *Plumeria pudica* Jacq. cv. Bridal Bouquet. |
| 65 | G76-4 | *Barringtonia asiatica* (L.) Kurz |
| 66 | S70-3 | *Passiflora x alato-caerulea* Lindl. |
| 67 | S40-4 | *Rosa damascena* Mill. |
| 68 | S58-3 | *Talauma candollei* Bl. |
| 69 | S74-3 | *Crinum asiaticum* Linn. |
| 70 | S74-4 | *Crinum asiaticum* Linn. |
