# Supplementary material for: Investigation of Non‐Saccharomyces Yeasts for Developing Unique Flavor Profiles in Nonalcoholic Mulberry Fermented Beverage
Source: Int J Food Sci. 2025 Sep 19;2025:5596446. doi: 10.1155/ijfo/5596446 (PMC12447106; doi:10.1155/ijfo/5596446)
Supplement: Supplementary file 2 — Supporting Information 2 Table S2: Identification of 36 isolated non‐Saccharomyces yeast strains based on the sequences of the D1/D2 domain of the 26S rDNA gene. [file IJFO-2025-5596446-s004.docx]

**Table S2.** Identification of 36 isolated non-*Saccharomyces* yeast strains based on the sequences of the D1/D2 domain of 26S rDNA gene.

| Strain | Closest Species  (Accession number) | Nucleotide identity  in D1/D2 domain | | Nucleotide different  in D1/D2 domain | | | Result of identification |
| --- | --- | --- | --- | --- | --- | --- | --- |
|  |  | No. nucleotides identity / total nucleotides | % identity | No. of Nucleotide substitutions | % substitutions | No. of gap |  |
| **Ascomycetous** | |  |  |  |  |  |  |
| S49-5 | *Hanseniaspora guilliermondii*  CBS 465 (KY107797) | 594/594 | 100 | 0 | 0 | 0 | *Hanseniaspora guilliermondii* |
| S61-2 | *Hanseniaspora guilliermondii*  CBS 465 (KY107797) | 604/604 | 100 | 0 | 0 | 0 | *Hanseniaspora guilliermondii* |
| S64-2 | *Hanseniaspora thailandica*  (nom. inval.) (DQ404527) | 593/594 | 99.83 | 1 | 0.2 | 0 | *Hanseniaspora thailandica* |
| 18-2 | *Hanseniaspora guilliermondii*  CBS 465 (KY107797) | 594/594 | 100 | 0 | 0 | 0 | *Hanseniaspora guilliermondii* |
| G62-2 | *Hanseniaspora opuntiae*  JW11-4 (KU316739) | 594/595 | 99.83 | 0 | 0 | 1 | *Hanseniaspora opuntiae* |
| G63-2 | *Hanseniaspora opuntiae*  JW11-4 (KU316739) | 603/603 | 100 | 0 | 0 | 0 | *Hanseniaspora opuntiae* |
| G77-2 | *Hanseniaspora opuntiae*  JW11-4 (KU316739) | 594/594 | 100 | 0 | 0 | 0 | *Hanseniaspora opuntiae* |
| S10-4 | *Nakaseomyces nivariensis*  CBS 9983 (MH545923) | 603/603 | 100 | 0 | 0 | 0 | *Nakaseomyces nivariensis* |
| S15-2 | *Candida tropicalis*  CBS 1920 (MH545915) | 602/602 | 100 | 0 | 0 | 0 | *Candida tropicalis* |
| S45-2 | *Meyerozyma caribbica*  CBS 9966 (MH545919) | 591/592 | 99.83 | 0 | 0 | 1 | *Meyerozyma caribbica* |
| S45-3 | *Meyerozyma caribbica*  CBS 9966 (MH545919) | 591/593 | 99.66 | 1 | 0.2 | 1 | *Meyerozyma caribbica* |
| S53-3 | *Meyerozyma caribbica*  CBS 9966 (MH545919) | 591/591 | 100 | 0 | 0 | 0 | *Meyerozyma caribbica* |
| S73-2 | *Meyerozyma caribbica*  CBS 9966 (MH545919) | 593/593 | 100 | 0 | 0 | 0 | *Meyerozyma caribbica* |
| S74-1 | *Nakaseomyces nivariensis* CBS 9983 (MH545923) | 602/603 | 99.83 | 1 | 0.2 | 0 | *Nakaseomyces nivariensis* |
| S11-4 | *Pichia kudriavzevii* CBS 5147 (MH545928) | 586/587 | 99.82 | 0 | 0 | 1 | *Pichia kudriavzevii* |
| S11-5 | *Pichia kudriavzevii* CBS 5147 (MH545928) | 587/588 | 99.82 | 0 | 0 | 1 | *Pichia kudriavzevii* |
| S14-5 (1) | *Pichia kudriavzevii* CBS 5147 (MH545928) | 586/586 | 100 | 0 | 0 | 0 | *Pichia kudriavzevii* |
| S14-5 (2) | *Pichia kudriavzevii* CBS 5147 (MH545928) | 586/586 | 100 | 0 | 0 | 0 | *Pichia kudriavzevii* |
| S62-3 | *Pichia kudriavzevii* CBS 5147 (MH545928) | 586/587 | 99.82 | 0 | 0 | 1 | *Pichia kudriavzevii* |
| S64-3 | *Pichia pijperi* NBRC 102058 (*Wickerhamomyces pijperi*) (AB449694) | 567/568 | 99.82 | 1 | 0.2 | 0 | *Pichia pijperi*  (*Wickerhamomyces pijperi*) |
| S76-1 | *Pichia kudriavzevii*  CBS 5147 (MH545928) | 586/587 | 99.82 | 0 | 0 | 1 | *Pichia kudriavzevii* |
| S76-3 | *Pichia kudriavzevii*  CBS 5147 (MH545928) | 586/586 | 100 | 0 | 0 | 1 | *Pichia kudriavzevii* |
| 7/4-2-4 | *Kodamaea ohmeri*  CBS 5367 (MK394144) | 527/527 | 100 | 0 | 0 | 0 | *Kodamaea ohmeri* |
| 7/4-3 | *Nakaseomyces nivariensis*  CBS 9983 (MH545923) | 613/613 | 100 | 0 | 0 | 0 | *Nakaseomyces nivariensis* |
| G27-1 | *Pichia kudriavzevii*  CBS 5147 (MH545928) | 587/588 | 99.82 | 0 | 0 | 1 | *Pichia kudriavzevii* |
| G8-5 (1) | *Pichia aff. fermentans* Y0_150  (LC015269) | 568/568 | 100 | 0 | 0 | 0 | *Pichia aff. fermentans* |
| G8-5 (2) | *Hanseniaspora opuntiae*  JW11-4 (KU316739) | 606/608 | 99.67 | 0 | 0 | 2 | *Hanseniaspora opuntiae* |
| G26-2 | *Kodamaea kakaduensis*  CBS 8611 (NG_058321) | 518/518 | 100 | 0 | 0 | 0 | *Kodamaea kakaduensis* |
| G51-1 | *Kodamaea ohmeri*  CBS 5367 (MK394144) | 525/525 | 100 | 0 | 0 | 0 | *Kodamaea ohmeri* |
| G76-4 | *Starmerella floricola*  CBS 7289 (KY106448) | 524/527 | 99.43 | 2 | 0.4 | 1 | *Starmerella floricola* |
| S70-3 | *Torulaspora pretoriensis*  CBS 11124 (KY109883) | 596/597 | 99.83 | 1 | 0.2 | 0 | *Torulaspora pretoriensis* |
| S40-4 | *Kurtzmaniella quercitrusa*  CBS 4412 (MK394107) | 586/587 | 99.82 | 1 | 0.2 | 0 | *Kurtzmaniella quercitrusa* |
| S74-3 | *Kodamaea ohmeri*  CBS 5367 (MK394144) | 516/516 | 100 | 0 | 0 | 0 | *Kodamaea ohmeri* |
| S74-4 | *Kodamaea ohmeri*  CBS 5367 (MK394144) | 515/515 | 100 | 0 | 0 | 0 | *Kodamaea ohmeri* |
| **Basidiomycetous** | |  |  |  |  |  |  |
| G23-8 (2) | *Moesziomyces aphidis* UZ271_17 (MF062260) | 623/623 | 100 | 0 | 0 | 0 | *Moesziomyces aphidis* |
| S58-3 | *Trichosporon asahii* strain 3.4-4 (FJ455110) | 628/628 | 100 | 0 | 0 | 0 | *Trichosporon asahii* |
