# Supplementary material for: Investigation of Non‐Saccharomyces Yeasts for Developing Unique Flavor Profiles in Nonalcoholic Mulberry Fermented Beverage
Source: Int J Food Sci. 2025 Sep 19;2025:5596446. doi: 10.1155/ijfo/5596446 (PMC12447106; doi:10.1155/ijfo/5596446)
Supplement: Supplementary file 3 — Supporting Information 3 Table S3: Concentrations (micrograms per liter) of volatile aroma compounds in wines fermented with different yeast strains and diluted mulberry juice (MJ). [file IJFO-2025-5596446-s003.docx]

**Table S3.** Concentrations (μg/L) of volatile aroma compounds in wines fermented with different yeast strains and diluted mulberry juice (MJ).

| Rt | Compounds | MJ (1:4) | S49-5 | S61-2 | S64-2 | 18-2 | G62-2 | G63-2 | G77-2 | S10-4 | S15-2 | S45-2 |
| --- | --- | --- | --- | --- | --- | --- | --- | --- | --- | --- | --- | --- |
| 6.235 | 1-Propanol, 2-methyl- (Isobutanol) | 0.00 | 1.63 | 1.50 | 8.01 | 17.36 | 19.75 | 22.15 | 23.55 | 2.39 | 38.58 | 8.47 |
| 8.788 | 1-Butanol, 3-methyl- (Isoamyl alcohol) | 2.46 | 12.90 | 18.65 | 61.20 | 132.58 | 160.01 | 123.00 | 225.46 | 19.46 | 195.48 | 58.41 |
| 12.342 | 1-Hexanol | 0.25 | 0.06 | 0.11 | 0.55 | 1.15 | 1.14 | 0.63 | 0.67 | 0.08 | 0.00 | 0.70 |
| 13.315 | 2-Hexanol, 2-methyl- | 0.00 | 0.55 | 0.00 | 6.21 | 6.59 | 6.97 | 0.00 | 6.48 | 0.00 | 2.99 | 7.43 |
| 15.431 | 2-Propyl-1-pentanol | 6.71 | 0.64 | 0.72 | 4.91 | 6.87 | 6.79 | 5.76 | 6.96 | 0.65 | 0.00 | 0.00 |
| 16.846 | 1-Octanol | 0.51 | 0.19 | 0.24 | 2.51 | 2.32 | 2.64 | 2.17 | 2.56 | 0.07 | 0.56 | 0.00 |
| 18.902 | 1-Undecanol | 0.00 | 0.32 | 0.44 | 3.25 | 3.95 | 3.72 | 3.39 | 3.67 | 0.04 | 0.00 | 0.12 |
| 19.526 | 9-Tetradecen-1-ol, (E)- | 0.00 | 0.00 | 0.00 | 0.46 | 0.00 | 0.97 | 0.44 | 0.67 | 0.00 | 0.00 | 0.00 |
| 22.929 | Benzyl alcohol (Phenylmethyl alcohol) | 0.00 | 0.67 | 0.69 | 9.00 | 9.95 | 7.46 | 5.36 | 7.77 | 0.00 | 0.00 | 0.00 |
| 23.516 | Phenylethyl Alcohol | 0.00 | 4.28 | 5.62 | 58.79 | 49.66 | 177.47 | 105.41 | 196.72 | 9.53 | 87.11 | 15.26 |
| 19.988 | 1-Propanol, 3-(methylthio)- | 0.00 | 0.00 | 0.00 | 0.00 | 0.00 | 0.00 | 0.00 | 0.00 | 0.10 | 0.00 | 0.00 |
| 15.424 | 1-Hexanol, 2-ethyl- | 0.00 | 0.00 | 0.00 | 0.00 | 0.00 | 0.00 | 0.00 | 0.00 | 0.00 | 6.44 | 6.47 |
| 17.685 | Terpinen-4-ol | 0.00 | 0.00 | 0.00 | 0.00 | 0.00 | 0.00 | 0.00 | 0.00 | 0.00 | 0.00 | 0.00 |
| 7.707 | Linalool | 0.00 | 0.00 | 0.00 | 0.00 | 0.00 | 0.00 | 0.00 | 0.00 | 0.00 | 0.00 | 0.00 |
| 20.840 | 1-Decanol | 0.00 | 0.00 | 0.00 | 0.00 | 0.00 | 0.00 | 0.00 | 0.00 | 0.00 | 0.00 | 0.00 |
|  | ∑ Alcohols | 9.93 | 21.23 | 27.97 | 154.89 | 230.43 | 386.94 | 268.31 | 474.51 | 32.33 | 331.17 | 96.86 |
| 14.589 | Dodecanal | 0.00 | 0.00 | 0.00 | 0.36 | 0.00 | 0.00 | 0.00 | 0.00 | 0.00 | 0.00 | 0.00 |
| 16.085 | Benzaldehyde | 1.34 | 0.00 | 0.00 | 1.38 | 0.00 | 2.21 | 1.61 | 2.48 | 0.21 | 0.00 | 0.58 |
| 1.661 | Acetaldehyde | 0.00 | 0.00 | 0.00 | 0.00 | 0.00 | 0.00 | 0.00 | 11.32 | 0.00 | 0.00 | 0.00 |
|  | ∑ Aldehydes | 1.34 | 0.00 | 0.00 | 1.75 | 0.00 | 2.21 | 1.61 | 13.80 | 0.21 | 0.00 | 0.58 |
| 10.669 | Acetoin | 0.00 | 0.17 | 0.22 | 1.65 | 1.81 | 2.30 | 2.64 | 3.07 | 0.30 | 0.00 | 0.00 |
| 16.333 | Oxacyclododecan-2-one | 0.23 | 0.00 | 0.00 | 0.00 | 0.00 | 0.00 | 0.00 | 0.00 | 0.00 | 0.00 | 0.00 |
| 17.413 | Isophorone | 0.95 | 0.28 | 0.36 | 2.89 | 2.94 | 1.73 | 1.34 | 0.71 | 0.19 | 1.30 | 2.00 |
| 25.468 | 2(3H)-Furanone, dihydro-5-pentyl- | 0.00 | 0.07 | 0.08 | 0.45 | 0.81 | 0.96 | 0.65 | 0.74 | 0.05 | 0.42 | 0.00 |
| 30.244 | Oxacyclododecan-2-one | 0.00 | 0.00 | 0.00 | 0.42 | 0.00 | 0.00 | 0.00 | 0.00 | 0.00 | 0.00 | 0.00 |
| 34.372 | 4-Acetoxy-3-methoxyacetophenone | 0.00 | 0.00 | 0.00 | 0.00 | 4.44 | 5.73 | 3.74 | 0.00 | 0.21 | 0.00 | 0.00 |
| 27.316 | 2(3H)-Furanone, 5-hexyldihydro- | 0.00 | 0.00 | 0.00 | 0.00 | 0.00 | 0.00 | 0.00 | 0.00 | 0.00 | 0.00 | 0.24 |
| 8.593 | 2-Octanone | 0.00 | 0.00 | 0.00 | 0.00 | 0.00 | 0.00 | 0.00 | 0.00 | 0.00 | 0.00 | 0.00 |
| 32.189 | Benzophenone | 0.00 | 0.00 | 0.00 | 0.00 | 0.00 | 0.00 | 0.00 | 0.00 | 0.00 | 0.00 | 0.00 |
|  | ∑ Ketones | 1.18 | 0.52 | 0.66 | 5.41 | 10.01 | 10.72 | 8.36 | 4.52 | 0.76 | 1.72 | 2.24 |
| 14.750 | Acetic acid | 0.00 | 0.63 | 1.64 | 11.53 | 8.42 | 5.90 | 8.75 | 6.72 | 0.48 | 1.90 | 1.08 |
| 17.181 | Propanoic acid, 2-methyl- (Isobutyric acid) | 0.00 | 0.25 | 0.27 | 0.74 | 1.73 | 3.64 | 2.56 | 4.70 | 0.10 | 1.05 | 0.65 |
| 19.197 | Butanoic acid, 2-methyl- (2-Methylbutyric acid) | 0.00 | 0.12 | 0.15 | 0.00 | 0.00 | 1.25 | 1.04 | 2.91 | 0.06 | 3.69 | 0.93 |
| 19.173 | Butanoic acid, 3-methyl- (Isovaleric acid) | 0.00 | 0.00 | 0.00 | 0.00 | 0.00 | 0.00 | 0.00 | 0.00 | 0.00 | 0.00 | 0.00 |
| 22.433 | 1-Hexanoic acid | 1.06 | 0.17 | 0.28 | 1.29 | 1.60 | 4.45 | 4.38 | 3.24 | 0.15 | 7.61 | 2.10 |
| 24.217 | Hexanoic acid, 2-ethyl- | 1.54 | 0.24 | 0.40 | 1.23 | 2.88 | 2.24 | 1.79 | 2.33 | 0.19 | 1.35 | 1.66 |
| 24.281 | Heptanoic acid | 0.34 | 0.00 | 0.00 | 0.00 | 0.00 | 0.00 | 0.00 | 0.00 | 0.00 | 0.59 | 0.57 |
| 26.048 | Octanoic acid | 1.26 | 3.68 | 6.48 | 17.40 | 30.30 | 81.73 | 90.20 | 80.99 | 1.27 | 61.99 | 9.74 |
| 27.732 | Nonanoic acid | 1.93 | 0.71 | 0.76 | 4.80 | 6.20 | 7.78 | 5.79 | 6.41 | 0.32 | 2.62 | 1.70 |
| 29.338 | n-Decanoic acid | 0.47 | 1.62 | 4.09 | 12.61 | 11.44 | 56.51 | 79.74 | 37.27 | 0.45 | 3.29 | 0.78 |
| 30.244 | Undecylenic acid | 0.0 | 0.29 | 0.08 | 0.00 | 3.20 | 5.28 | 3.54 | 10.45 | 0.05 | 0.00 | 0.00 |
| 31.635 | Benzoic acid | 0.00 | 0.10 | 0.12 | 0.71 | 1.18 | 1.21 | 1.04 | 1.49 | 0.00 | 0.42 | 0.27 |
| 32.360 | Dodecanoic acid | 0.0 | 0.13 | 0.27 | 0.87 | 0.60 | 2.77 | 5.90 | 1.70 | 0.00 | 0.00 | 0.00 |
| 18.379 | Butanoic acid | 0.0 | 0.00 | 0.00 | 0.00 | 0.00 | 0.00 | 0.00 | 0.00 | 0.00 | 0.67 | 0.25 |
|  | ∑ Acids | 6.60 | 7.94 | 14.54 | 51.19 | 67.56 | 172.76 | 204.75 | 158.20 | 3.05 | 85.17 | 19.72 |
| 2.379 | Ethyl Acetate | 9.83 | 31.59 | 65.04 | 383.70 | 337.98 | 217.39 | 330.31 | 343.24 | 1.42 | 8.32 | 4.22 |
| 6.440 | 1-Butanol, 3-methyl-, acetate (Isoamyl acetate) | 0 | 3.94 | 8.43 | 677.00 | 38.83 | 26.29 | 38.93 | 37.52 | 1.09 | 0.00 | 0.00 |
| 18.104 | Benzoic acid, methyl ester (Methyl benzoate) | 0.82 | 0 | 0 | 0 | 0 | 0 | 0 | 0 | 0.07 | 0.77 | 0.60 |
| 18.989 | Benzoic acid, ethyl ester (Ethyl benzoate) | 1.97 | 0 | 0 | 0 | 0 | 0 | 0 | 0 | 0 | 0 | 0 |
| 20.223 | Acetic acid, phenylmethyl ester (Benzyl acetate) | 0 | 0.23 | 0.36 | 5.70 | 1.64 | 0.00 | 0.84 | 0.43 | 0.00 | 0.00 | 0.00 |
| 21.035 | Methyl salicylate | 0.70 | 0.42 | 0.54 | 11.82 | 7.89 | 11.45 | 6.77 | 22.06 | 0.22 | 1.44 | 0.70 |
| 33.104 | 1,2-Benzenedicarboxylic acid, butyl octyl ester | 0 | 0 | 0.00 | 0.29 | 0.00 | 0.47 | 0.38 | 0.00 | 0.00 | 0.00 | 0.00 |
| 21.286 | Hydrazinecarboxylic acid, phenylmethyl ester | 0.00 | 0 | 0.00 | 0 | 0 | 0 | 0.84 | 0.00 | 0.00 | 0.00 | 0.00 |
| 14.203 | Octanoic acid, ethyl ester (Ethyl octanoate) | 0.00 | 0 | 0.00 | 0 | 0 | 0 | 0.00 | 0.00 | 0 | 0 | 0 |
| 35.247 | Dibutyl phthalate (Phthalic acid, dibutyl ester) | 0 | 0.08 | 0.12 | 0.61 | 1.03 | 0.91 | 0.78 | 0.88 | 0.05 | 0.00 | 0.22 |
|  | ∑ Esters | 13.32 | 36.26 | 74.50 | 1079.11 | 387.37 | 256.51 | 378.85 | 404.13 | 2.84 | 10.52 | 5.75 |
| 1.674 | 2-Octanamine | 0.00 | 0.00 | 0.00 | 6.68 | 0.00 | 0.00 | 13.08 | 0.00 | 0.00 | 0.00 | 0.00 |
| 9.871 | Cyclohexene, 1-methyl-4-(1-methylethylidene)- (Terpinolene) | 0 | 0 | 0 | 1.94 | 0.00 | 0.00 | 0.00 | 1.08 | 0.00 | 1.54 | 0.00 |
| 14.670 | Cycloheptane | 0.00 | 0.00 | 0.00 | 0.00 | 0.00 | 1.43 | 1.45 | 1.58 | 0.00 | 0.00 | 0.50 |
| 10.095 | p-Cymene | 0.38 | 0.11 | 0.06 | 0.00 | 0.00 | 0.00 | 1.97 | 0.00 | 0.11 | 1.66 | 0.83 |
| 10.203 | o-Cymene | 1.41 | 0.25 | 0.36 | 2.90 | 3.88 | 3.43 | 0.82 | 0.98 | 0.10 | 0.73 | 1.21 |
| 10.085 | Benzene, 1-methyl-3-(1-methylethyl)- | 0 | 0 | 0 | 0 | 0 | 0 | 0 | 1.50 | 0.00 | 0 | 0.00 |
| 9.864 | Styrene | 0 | 0 | 0 | 0 | 0 | 0 | 0 | 0 | 0 | 0 | 0 |
|  | ∑ Hydrocarbons | 1.79 | 0.35 | 0.42 | 11.52 | 3.88 | 4.87 | 17.32 | 5.15 | 0.21 | 3.93 | 2.54 |
| 26.451 | p-Cresol | 0 | 0.08 | 0.10 | 0.83 | 1.07 | 1.02 | 0.93 | 1.25 | 0.00 | 0.00 | 0.00 |
| 28.480 | Phenol, 2-methyl-5-(1-methylethyl)- (Carvacrol) | 0.28 | 0.58 | 0.71 | 5.23 | 9.47 | 6.79 | 4.53 | 7.04 | 0.05 | 0.46 | 0.33 |
| 29.969 | 2,4-Di-tert-butylphenol | 2.67 | 1.41 | 1.63 | 10.03 | 14.25 | 12.70 | 14.20 | 12.29 | 0.83 | 6.97 | 4.28 |
| 28.057 | Thymol | 0 | 0 | 0 | 0.34 | 0.00 | 0 | 0 | 0.00 | 0.00 | 0 | 0.00 |
| 24.375 | Phenol, 2,6-dimethyl- | 0 | 0 | 0 | 0 | 0 | 0 | 0 | 0 | 0.05 | 0 | 0.00 |
| 25.532 | Phenol, 2-methoxy-4-propyl- (Propyl guaiacol) | 0 | 0 | 0 | 0 | 0 | 0 | 0 | 0 | 0 | 0 | 1.74 |
| 27.896 | Phenol, 4-ethyl- | 0 | 0 | 0 | 0 | 0 | 0 | 0 | 0 | 0 | 0 | 1.02 |
| 28.181 | Phenol, 2,3,5,6-tetramethyl- | 0 | 0 | 0 | 0 | 0 | 0 | 0 | 0 | 0 | 0 | 0 |
| 17.121 | 1,3-Benzenediol, 4-ethyl- (Resorcinol) | 0.26 | 0 | 0 | 0 | 0 | 0 | 0 | 0 | 0 | 0 | 0 |
|  | ∑ Phenols | 3.21 | 2.07 | 2.44 | 16.43 | 24.79 | 20.51 | 19.66 | 20.57 | 0.92 | 7.44 | 7.38 |
| 18.610 | 2-Toluic hydrazide | 0 | 0 | 0 | 5.59 | 0.00 | 0.00 | 0.00 | 0.00 | 0.00 | 0.00 | 6.13 |
| 18.607 | Phthalan | 12.81 | 0.74 | 0.73 | 0.00 | 6.59 | 8.76 | 6.34 | 9.79 | 0.21 | 8.71 | 0.00 |
| 16.145 | 2-Thiazolidinimine, 3-methyl- | 0 | 0 | 0 | 0 | 0 | 0 | 0 | 0 | 0 | 0 | 0 |
| 21.280 | Benzyl 2-chloroethyl sulfone | 0 | 0 | 0 | 0 | 0 | 0 | 0 | 0 | 0 | 0 | 0 |
|  | ∑ Miscellaneous | 12.81 | 0.74 | 0.73 | 5.59 | 6.59 | 8.76 | 6.34 | 9.79 | 0.21 | 8.71 | 6.13 |

**Table S3.** (continued).

| Rt | Compounds | S45-3 | S53-3 | S73-2 | S74-1 | S11-4 | S11-5 | S14-5(1) | S14-5(2) | S62-3 | S64-3 | S76-1 |
| --- | --- | --- | --- | --- | --- | --- | --- | --- | --- | --- | --- | --- |
| 6.235 | 1-Propanol, 2-methyl- (Isobutanol) | 7.20 | 6.19 | 6.60 | 44.65 | 0.74 | 34.08 | 41.60 | 29.60 | 34.46 | 10.63 | 35.53 |
| 8.788 | 1-Butanol, 3-methyl- (Isoamyl alcohol) | 62.78 | 57.40 | 62.49 | 250.25 | 171.80 | 186.40 | 192.72 | 179.27 | 197.35 | 110.54 | 208.79 |
| 12.342 | 1-Hexanol | 0.64 | 0.79 | 0.80 | 1.09 | 0.00 | 0.00 | 0.00 | 0.00 | 0.00 | 0.89 | 0.00 |
| 13.315 | 2-Hexanol, 2-methyl- | 6.35 | 7.05 | 6.30 | 6.31 | 7.11 | 0.00 | 0.00 | 0.00 | 6.72 | 0.00 | 6.97 |
| 15.431 | 2-Propyl-1-pentanol | 0.00 | 6.52 | 0.00 | 0.00 | 0.00 | 0.00 | 0.00 | 0.00 | 0.00 | 0.00 | 0.00 |
| 16.846 | 1-Octanol | 0.00 | 0.37 | 0.00 | 1.02 | 1.01 | 0.96 | 1.14 | 1.16 | 0.89 | 1.14 | 1.01 |
| 18.902 | 1-Undecanol | 0.00 | 0.21 | 0.19 | 0.40 | 0.76 | 0.71 | 0.88 | 0.87 | 0.70 | 0.93 | 0.78 |
| 19.526 | 9-Tetradecen-1-ol, (E)- | 0.00 | 0.00 | 0.00 | 0.00 | 0.00 | 0.00 | 0.00 | 0.00 | 0.00 | 0.00 | 0.00 |
| 22.929 | Benzyl alcohol (Phenylmethyl alcohol) | 0.00 | 0.00 | 0.00 | 0.00 | 0.00 | 0.00 | 0.00 | 0.00 | 0.00 | 0.00 | 0.00 |
| 23.516 | Phenylethyl Alcohol | 18.94 | 17.56 | 17.78 | 115.62 | 114.59 | 115.59 | 136.50 | 117.57 | 116.46 | 51.35 | 123.60 |
| 19.988 | 1-Propanol, 3-(methylthio)- | 0.00 | 0.00 | 0.00 | 0.00 | 0.00 | 0.00 | 0.00 | 0.00 | 0.00 | 0.00 | 0.00 |
| 15.424 | 1-Hexanol, 2-ethyl- | 6.12 | 0.00 | 7.08 | 7.18 | 7.30 | 7.64 | 6.62 | 7.24 | 6.77 | 6.96 | 7.52 |
| 17.685 | Terpinen-4-ol | 0.00 | 0.00 | 0.00 | 0.00 | 0.00 | 0.00 | 0.00 | 0.00 | 0.00 | 0.00 | 0.00 |
| 7.707 | Linalool | 0.00 | 0.00 | 0.00 | 0.00 | 0.00 | 0.00 | 0.00 | 0.00 | 0.00 | 0.00 | 0.00 |
| 20.840 | 1-Decanol | 0.00 | 0.00 | 0.00 | 0.00 | 0.00 | 0.00 | 0.00 | 0.00 | 0.00 | 0.00 | 0.00 |
|  | ∑ Alcohols | 102.03 | 96.09 | 101.26 | 426.53 | 303.31 | 345.38 | 379.46 | 335.72 | 363.36 | 182.43 | 384.20 |
| 14.589 | Dodecanal | 0.00 | 0.00 | 0.00 | 0.00 | 0.00 | 0.00 | 0.00 | 0.00 | 0.00 | 0.00 | 0.00 |
| 16.085 | Benzaldehyde | 0.48 | 0.00 | 0.00 | 3.02 | 0.00 | 0.00 | 1.82 | 0.00 | 0.00 | 0.00 | 0.00 |
| 1.661 | Acetaldehyde | 0.00 | 0.00 | 0.00 | 0.00 | 0.00 | 0.00 | 0.00 | 0.00 | 0.00 | 0.00 | 0.00 |
|  | ∑ Aldehydes | 0.48 | 0.00 | 0.00 | 3.02 | 0.00 | 0.00 | 1.82 | 0.00 | 0.00 | 0.00 | 0.00 |
| 10.669 | Acetoin | 0.00 | 0.00 | 0.00 | 2.86 | 0.00 | 0.00 | 0.00 | 0.00 | 0.00 | 0.00 | 0.00 |
| 16.333 | Oxacyclododecan-2-one | 0.00 | 0.00 | 0.00 | 0.00 | 0.00 | 0.00 | 0.00 | 0.00 | 0.00 | 0.00 | 0.00 |
| 17.413 | Isophorone | 1.46 | 2.33 | 2.15 | 1.53 | 1.97 | 1.33 | 1.45 | 0.00 | 0.87 | 0.83 | 1.13 |
| 25.468 | 2(3H)-Furanone, dihydro-5-pentyl- | 0.11 | 0.00 | 0.00 | 0.69 | 0.00 | 0.67 | 0.64 | 0.75 | 0.75 | 0.00 | 0.83 |
| 30.244 | Oxacyclododecan-2-one | 0.00 | 0.00 | 0.00 | 0.00 | 0.00 | 0.00 | 0.00 | 0.00 | 0.00 | 0.00 | 0.00 |
| 34.372 | 4-Acetoxy-3-methoxyacetophenone | 0.00 | 0.00 | 0.00 | 0.00 | 0.00 | 0.00 | 0.00 | 0.00 | 0.00 | 0.00 | 0.00 |
| 27.316 | 2(3H)-Furanone, 5-hexyldihydro- | 0.21 | 0.52 | 0.00 | 0.00 | 0.00 | 0.00 | 0.00 | 0.00 | 0.00 | 0.00 | 0.00 |
| 8.593 | 2-Octanone | 0.00 | 0.00 | 0.00 | 0.00 | 0.00 | 0.00 | 0.00 | 0.00 | 0.00 | 0.00 | 0.00 |
| 32.189 | Benzophenone | 0.00 | 0.00 | 0.00 | 0.00 | 0.00 | 0.00 | 0.00 | 0.00 | 0.00 | 0.00 | 0.00 |
|  | ∑ Ketones | 1.78 | 2.86 | 2.15 | 5.08 | 1.97 | 2.00 | 2.09 | 0.75 | 1.62 | 0.83 | 1.95 |
| 14.750 | Acetic acid | 1.24 | 1.07 | 0.95 | 3.83 | 2.89 | 2.65 | 5.01 | 2.89 | 2.77 | 12.68 | 3.30 |
| 17.181 | Propanoic acid, 2-methyl- (Isobutyric acid) | 0.66 | 0.35 | 0.42 | 0.88 | 5.24 | 5.25 | 12.43 | 5.19 | 4.30 | 0.70 | 5.28 |
| 19.197 | Butanoic acid, 2-methyl- (2-Methylbutyric acid) | 0.98 | 0.75 | 0.75 | 0.00 | 0.00 | 0.00 | 0.00 | 0.00 | 0.00 | 0.97 | 0.00 |
| 19.173 | Butanoic acid, 3-methyl- (Isovaleric acid) | 0.00 | 0.00 | 0.00 | 0.00 | 16.99 | 17.82 | 15.30 | 15.26 | 12.78 | 0.00 | 16.94 |
| 22.433 | 1-Hexanoic acid | 2.24 | 1.46 | 1.45 | 1.68 | 2.15 | 2.11 | 2.45 | 1.97 | 2.10 | 0.30 | 2.37 |
| 24.217 | Hexanoic acid, 2-ethyl- | 1.63 | 2.87 | 1.23 | 3.27 | 1.78 | 1.90 | 2.87 | 1.55 | 1.68 | 2.07 | 2.34 |
| 24.281 | Heptanoic acid | 0.51 | 0.23 | 0.34 | 0.00 | 0.94 | 0.99 | 0.97 | 0.88 | 0.89 | 0.00 | 0.95 |
| 26.048 | Octanoic acid | 10.67 | 5.55 | 6.16 | 8.34 | 15.48 | 15.18 | 23.58 | 15.39 | 18.86 | 1.52 | 18.39 |
| 27.732 | Nonanoic acid | 1.52 | 1.60 | 1.46 | 3.19 | 2.80 | 2.76 | 2.52 | 2.64 | 2.67 | 1.40 | 2.75 |
| 29.338 | n-Decanoic acid | 0.92 | 0.56 | 0.61 | 2.40 | 1.98 | 1.90 | 1.84 | 1.62 | 2.15 | 0.51 | 2.18 |
| 30.244 | Undecylenic acid | 0.00 | 0.00 | 0.00 | 0.00 | 0.00 | 0.00 | 0.00 | 0.00 | 0.00 | 0.00 | 0.00 |
| 31.635 | Benzoic acid | 0.24 | 0.24 | 0.25 | 0.36 | 0.00 | 0.00 | 0.57 | 0.00 | 0.00 | 0.33 | 0.00 |
| 32.360 | Dodecanoic acid | 0.00 | 0.00 | 0.00 | 0.00 | 0.00 | 0.00 | 0.00 | 0.00 | 0.00 | 0.00 | 0.00 |
| 18.379 | Butanoic acid | 0.30 | 0.00 | 0.00 | 0.00 | 0.00 | 0.00 | 0.00 | 0.00 | 0.00 | 0.00 | 0.00 |
|  | ∑ Acids | 20.90 | 14.67 | 13.63 | 23.95 | 50.25 | 50.55 | 67.55 | 47.39 | 48.21 | 20.48 | 54.51 |
| 2.379 | Ethyl Acetate | 4.34 | 4.58 | 5.74 | 12.77 | 572.01 | 573.36 | 221.68 | 597.27 | 599.46 | 545.39 | 666.79 |
| 6.440 | 1-Butanol, 3-methyl-, acetate (Isoamyl acetate) | 0.00 | 0.00 | 0.00 | 8.81 | 41.42 | 31.84 | 8.37 | 24.56 | 23.44 | 20.19 | 27.82 |
| 18.104 | Benzoic acid, methyl ester (Methyl benzoate) | 0.58 | 0.00 | 0.63 | 0.98 | 0.70 | 0.69 | 0.00 | 1.28 | 1.14 | 1.25 | 1.29 |
| 18.989 | Benzoic acid, ethyl ester (Ethyl benzoate) | 0 | 0.69 | 0 | 0 | 0 | 0 | 0 | 0 | 0 | 0 | 0 |
| 20.223 | Acetic acid, phenylmethyl ester (Benzyl acetate) | 0.00 | 0.00 | 0.00 | 0.00 | 0.00 | 0.00 | 0.00 | 0.00 | 0.00 | 0.00 | 0.00 |
| 21.035 | Methyl salicylate | 0.68 | 0.00 | 0.76 | 2.09 | 1.41 | 1.33 | 1.22 | 1.39 | 1.36 | 2.83 | 1.48 |
| 33.104 | 1,2-Benzenedicarboxylic acid, butyl octyl ester | 0.00 | 0.63 | 0.00 | 0.00 | 0.00 | 0.00 | 0.00 | 0.00 | 0.00 | 0.00 | 0.00 |
| 21.286 | Hydrazinecarboxylic acid, phenylmethyl ester | 0.00 | 0 | 0.00 | 0.00 | 0.89 | 0.92 | 0.00 | 0.00 | 0.78 | 0.00 | 0.00 |
| 14.203 | Octanoic acid, ethyl ester (Ethyl octanoate) | 0 | 0 | 0 | 0 | 0 | 0 | 0 | 0 | 0 | 0 | 0 |
| 35.247 | Dibutyl phthalate (Phthalic acid, dibutyl ester) | 0.60 | 0.24 | 0.23 | 0.50 | 0.00 | 0.00 | 0.00 | 0.00 | 0.00 | 0.27 | 0.00 |
|  | ∑ Esters | 6.20 | 6.15 | 7.36 | 25.16 | 616.43 | 608.15 | 231.27 | 624.49 | 626.19 | 569.94 | 697.38 |
| 1.674 | 2-Octanamine | 0.00 | 0.00 | 0.00 | 0.00 | 0.00 | 0.00 | 0.00 | 0.00 | 0.00 | 0.00 | 0.00 |
| 9.871 | Cyclohexene, 1-methyl-4-(1-methylethylidene)- (Terpinolene) | 0.00 | 0.00 | 0.89 | 0.00 | 0.00 | 0.00 | 0.00 | 0.00 | 0.00 | 0.00 | 0.00 |
| 14.670 | Cycloheptane | 6.07 | 0.84 | 0.00 | 0.00 | 0.00 | 0.00 | 0.00 | 0.67 | 0.00 | 0.00 | 0.00 |
| 10.095 | p-Cymene | 0.00 | 1.35 | 1.84 | 2.32 | 0.00 | 0.00 | 0.00 | 0.00 | 0.00 | 1.05 | 0.00 |
| 10.203 | o-Cymene | 0.75 | 0.68 | 0.75 | 0.88 | 0.00 | 0.00 | 3.23 | 0.00 | 0.00 | 1.31 | 0.00 |
| 10.085 | Benzene, 1-methyl-3-(1-methylethyl)- | 1.61 | 0.00 | 0.00 | 0.00 | 0.00 | 0.00 | 0.00 | 0.00 | 0.00 | 0.00 | 0.00 |
| 9.864 | Styrene | 0 | 0 | 0 | 0 | 0 | 0 | 0 | 0 | 0 | 0 | 0 |
|  | ∑ Hydrocarbons | 8.42 | 2.88 | 3.48 | 3.20 | 0.00 | 0.00 | 3.23 | 0.67 | 0.00 | 2.35 | 0.00 |
| 26.451 | p-Cresol | 0.19 | 0.09 | 0.00 | 0.00 | 0.00 | 0.00 | 0.00 | 0.00 | 0.00 | 0.00 | 0.00 |
| 28.480 | Phenol, 2-methyl-5-(1-methylethyl)- (Carvacrol) | 0.32 | 0.32 | 0.32 | 0.52 | 0.00 | 0.00 | 0.48 | 0.00 | 0.00 | 0.59 | 0.00 |
| 29.969 | 2,4-Di-tert-butylphenol | 4.21 | 3.87 | 4.24 | 7.21 | 6.40 | 5.58 | 6.12 | 5.91 | 6.35 | 5.31 | 6.32 |
| 28.057 | Thymol | 0.00 | 0.00 | 0.00 | 0.00 | 0.00 | 0.00 | 0.00 | 0.00 | 0.00 | 0.00 | 0.00 |
| 24.375 | Phenol, 2,6-dimethyl- | 0.00 | 0.24 | 0.00 | 0.63 | 0.69 | 0.73 | 0.67 | 1.51 | 1.21 | 0.62 | 1.45 |
| 25.532 | Phenol, 2-methoxy-4-propyl- (Propyl guaiacol) | 1.57 | 1.44 | 1.91 | 0 | 0.00 | 0.00 | 0.00 | 0.00 | 0.00 | 0.00 | 0 |
| 27.896 | Phenol, 4-ethyl- | 0.90 | 0.88 | 0.97 | 0 | 0.00 | 0.00 | 0.00 | 0.00 | 0.00 | 0.00 | 0 |
| 28.181 | Phenol, 2,3,5,6-tetramethyl- | 0 | 0 | 0 | 0 | 0 | 0 | 0 | 0 | 0 | 0 | 0 |
| 17.121 | 1,3-Benzenediol, 4-ethyl- (Resorcinol) | 0 | 0 | 0 | 0 | 0 | 0 | 0 | 0 | 0 | 0 | 0 |
|  | ∑ Phenols | 7.19 | 6.85 | 7.44 | 8.35 | 7.09 | 6.30 | 7.27 | 7.42 | 7.56 | 6.52 | 7.76 |
| 18.610 | 2-Toluic hydrazide | 0.00 | 0.00 | 0.00 | 0.00 | 0.00 | 0.00 | 0.00 | 0.00 | 0.00 | 0.00 | 0.00 |
| 18.607 | Phthalan | 0.19 | 3.57 | 3.74 | 2.57 | 2.15 | 2.12 | 3.08 | 2.80 | 2.84 | 5.29 | 3.50 |
| 16.145 | 2-Thiazolidinimine, 3-methyl- | 0 | 0 | 0 | 0 | 0 | 0 | 0 | 0 | 0 | 0 | 0 |
| 21.280 | Benzyl 2-chloroethyl sulfone | 0 | 0 | 0 | 0 | 0 | 0 | 1.97 | 0.00 | 0.00 | 0.00 | 0 |
|  | ∑ Miscellaneous | 0.19 | 3.57 | 3.74 | 2.57 | 2.15 | 2.12 | 5.05 | 2.80 | 2.84 | 5.29 | 3.50 |

**Table S3.** (continued).

| Rt | Compounds | S76-3 | 7/4-2-4 | 7/4-3 | G27-1 | G8-5(1) | G8-5(2) | G23-8(2) | G26-2 | G51-1 | G76-4 | S70-3 |
| --- | --- | --- | --- | --- | --- | --- | --- | --- | --- | --- | --- | --- |
| 6.235 | 1-Propanol, 2-methyl- (Isobutanol) | 38.23 | 12.42 | 16.11 | 16.02 | 9.17 | 10.53 | 0.00 | 7.03 | 11.25 | 1.96 | 21.65 |
| 8.788 | 1-Butanol, 3-methyl- (Isoamyl alcohol) | 208.64 | 88.36 | 187.65 | 83.68 | 88.50 | 139.70 | 3.19 | 68.50 | 82.90 | 8.33 | 220.69 |
| 12.342 | 1-Hexanol | 0.00 | 0.00 | 0.75 | 0.00 | 0.58 | 1.02 | 0.98 | 0.40 | 0.57 | 0.93 | 0.68 |
| 13.315 | 2-Hexanol, 2-methyl- | 0.00 | 0.00 | 6.37 | 0.00 | 6.46 | 8.24 | 0.00 | 6.03 | 1.30 | 6.55 | 6.65 |
| 15.431 | 2-Propyl-1-pentanol | 0.00 | 0.00 | 0.00 | 0.00 | 0.00 | 0.00 | 0.00 | 6.83 | 0.00 | 0.00 | 0.00 |
| 16.846 | 1-Octanol | 1.16 | 0.00 | 1.00 | 0.00 | 1.09 | 2.74 | 0.45 | 0.00 | 0.00 | 0.47 | 2.42 |
| 18.902 | 1-Undecanol | 0.81 | 0.00 | 0.42 | 0.00 | 0.35 | 3.36 | 0.00 | 0.00 | 0.00 | 0.00 | 2.51 |
| 19.526 | 9-Tetradecen-1-ol, (E)- | 0.00 | 0.00 | 0.00 | 0.00 | 0.00 | 1.32 | 0.00 | 0.00 | 0.00 | 0.29 | 0.48 |
| 22.929 | Benzyl alcohol (Phenylmethyl alcohol) | 0.00 | 0.00 | 0.00 | 0.00 | 0.00 | 9.63 | 0.00 | 0.41 | 0.00 | 0.00 | 0.00 |
| 23.516 | Phenylethyl Alcohol | 138.85 | 54.51 | 81.50 | 64.69 | 112.89 | 162.99 | 0.85 | 7.39 | 37.66 | 9.47 | 132.70 |
| 19.988 | 1-Propanol, 3-(methylthio)- | 0.00 | 0.24 | 0.00 | 0.00 | 0.00 | 0.00 | 0.00 | 0.00 | 0.00 | 0.00 | 0.00 |
| 15.424 | 1-Hexanol, 2-ethyl- | 7.46 | 6.52 | 7.18 | 10.38 | 7.06 | 6.58 | 5.74 | 0.00 | 6.29 | 5.98 | 6.00 |
| 17.685 | Terpinen-4-ol | 0.00 | 0.00 | 0.27 | 0.00 | 0.00 | 0.36 | 0.00 | 0.00 | 0.00 | 0.00 | 0.00 |
| 7.707 | Linalool | 0.00 | 0.00 | 0.00 | 0.00 | 0.00 | 2.19 | 0.00 | 0.00 | 0.00 | 1.01 | 0.00 |
| 20.840 | 1-Decanol | 0.00 | 0.00 | 0.00 | 0.00 | 0.00 | 0.00 | 0.00 | 0.00 | 0.00 | 0.00 | 0.00 |
|  | ∑ Alcohols | 395.15 | 162.05 | 301.26 | 174.78 | 226.10 | 348.66 | 11.21 | 96.60 | 139.97 | 34.99 | 393.77 |
| 14.589 | Dodecanal | 0.00 | 0.00 | 0.00 | 0.00 | 0.00 | 0.00 | 0.00 | 0.00 | 0.00 | 0.00 | 0.00 |
| 16.085 | Benzaldehyde | 1.50 | 1.28 | 4.46 | 0.00 | 0.71 | 3.89 | 1.00 | 0.00 | 0.80 | 1.51 | 0.65 |
| 1.661 | Acetaldehyde | 0.00 | 0.00 | 0.00 | 0.00 | 0.00 | 0.00 | 0.00 | 0.00 | 0.00 | 0.00 | 0.00 |
|  | ∑ Aldehydes | 1.50 | 1.28 | 4.46 | 0.00 | 0.71 | 3.89 | 1.00 | 0.00 | 0.80 | 1.51 | 0.65 |
| 10.669 | Acetoin | 0.00 | 2.83 | 2.39 | 0.00 | 0.00 | 1.73 | 0.00 | 5.60 | 2.00 | 1.04 | 0.00 |
| 16.333 | Oxacyclododecan-2-one | 0.00 | 0.00 | 0.00 | 0.00 | 0.00 | 0.00 | 0.00 | 0.00 | 0.00 | 0.00 | 0.00 |
| 17.413 | Isophorone | 1.89 | 0.00 | 0.68 | 1.45 | 1.67 | 1.26 | 0.00 | 1.39 | 2.14 | 0.74 | 2.41 |
| 25.468 | 2(3H)-Furanone, dihydro-5-pentyl- | 0.84 | 0.27 | 0.56 | 1.23 | 0.50 | 0.64 | 0.67 | 0.22 | 0.21 | 0.00 | 0.73 |
| 30.244 | Oxacyclododecan-2-one | 0.00 | 0.00 | 0.00 | 0.00 | 0.00 | 0.00 | 0.00 | 0.00 | 0.00 | 0.00 | 0.00 |
| 34.372 | 4-Acetoxy-3-methoxyacetophenone | 0.00 | 0.00 | 0.00 | 0.00 | 0.00 | 0.00 | 1.19 | 0.00 | 0.00 | 0.00 | 0.00 |
| 27.316 | 2(3H)-Furanone, 5-hexyldihydro- | 0.00 | 0.00 | 0.00 | 0.00 | 0.00 | 0.00 | 0.00 | 0.30 | 0.00 | 0.00 | 0.00 |
| 8.593 | 2-Octanone | 0.00 | 0.00 | 0.00 | 0.00 | 0.00 | 0.00 | 0.00 | 1.27 | 0.00 | 1.19 | 0.00 |
| 32.189 | Benzophenone | 0.00 | 0.00 | 0.00 | 0.00 | 0.00 | 0.00 | 0.00 | 0.00 | 0.00 | 0.00 | 0.00 |
|  | ∑ Ketones | 2.73 | 3.10 | 3.63 | 2.68 | 2.17 | 3.64 | 1.86 | 8.78 | 4.35 | 2.97 | 3.14 |
| 14.750 | Acetic acid | 3.63 | 1.87 | 3.95 | 0.00 | 8.32 | 4.18 | 0.00 | 5.11 | 2.78 | 2.11 | 1.31 |
| 17.181 | Propanoic acid, 2-methyl- (Isobutyric acid) | 6.54 | 0.42 | 0.00 | 4.36 | 3.25 | 3.15 | 0.00 | 0.56 | 0.57 | 0.00 | 5.01 |
| 19.197 | Butanoic acid, 2-methyl- (2-Methylbutyric acid) | 0.00 | 0.70 | 0.00 | 0.00 | 0.00 | 1.97 | 0.00 | 1.11 | 0.56 | 0.00 | 5.30 |
| 19.173 | Butanoic acid, 3-methyl- (Isovaleric acid) | 14.82 | 0.00 | 0.00 | 8.58 | 3.16 | 0.00 | 0.00 | 0.00 | 0.00 | 0.00 | 0.00 |
| 22.433 | 1-Hexanoic acid | 1.36 | 0.83 | 2.06 | 0.00 | 1.94 | 2.72 | 0.19 | 0.56 | 1.04 | 1.38 | 4.95 |
| 24.217 | Hexanoic acid, 2-ethyl- | 1.78 | 1.40 | 1.81 | 0.00 | 1.69 | 1.25 | 1.18 | 0.86 | 1.01 | 1.04 | 2.23 |
| 24.281 | Heptanoic acid | 0.85 | 0.35 | 0.00 | 0.00 | 0.82 | 0.00 | 0.00 | 0.25 | 0.30 | 0.49 | 0.00 |
| 26.048 | Octanoic acid | 11.34 | 2.00 | 13.22 | 24.37 | 20.56 | 37.31 | 0.44 | 0.90 | 2.40 | 1.26 | 50.18 |
| 27.732 | Nonanoic acid | 2.80 | 1.26 | 2.91 | 3.87 | 2.23 | 4.83 | 0.87 | 0.98 | 1.09 | 0.65 | 1.22 |
| 29.338 | n-Decanoic acid | 2.10 | 0.42 | 5.02 | 3.56 | 1.48 | 17.40 | 0.19 | 0.24 | 0.48 | 0.29 | 3.62 |
| 30.244 | Undecylenic acid | 0.00 | 0.00 | 0.26 | 0.00 | 0.00 | 2.47 | 0.00 | 0.00 | 0.00 | 0.00 | 0.81 |
| 31.635 | Benzoic acid | 0.59 | 0.35 | 0.37 | 0.86 | 0.52 | 0.93 | 0.17 | 0.30 | 0.31 | 0.23 | 0.55 |
| 32.360 | Dodecanoic acid | 0.00 | 0.00 | 0.36 | 0.00 | 0.00 | 1.61 | 0.00 | 0.00 | 0.00 | 0.00 | 0.00 |
| 18.379 | Butanoic acid | 0.00 | 0.00 | 0.00 | 0.00 | 0.00 | 0.00 | 0.00 | 0.00 | 0.00 | 0.00 | 0.00 |
|  | ∑ Acids | 45.79 | 9.60 | 29.96 | 45.59 | 43.97 | 77.83 | 3.03 | 10.86 | 10.54 | 7.45 | 75.19 |
| 2.379 | Ethyl Acetate | 730.22 | 2.23 | 11.82 | 800.63 | 7.15 | 281.07 | 3.44 | 0.00 | 0.93 | 5.87 | 15.56 |
| 6.440 | 1-Butanol, 3-methyl-, acetate (Isoamyl acetate) | 21.61 | 0.00 | 6.81 | 16.50 | 0.00 | 13.30 | 0.00 | 0.00 | 0.00 | 0.00 | 0.00 |
| 18.104 | Benzoic acid, methyl ester (Methyl benzoate) | 1.26 | 0.00 | 1.46 | 0.00 | 1.55 | 1.38 | 0.85 | 1.04 | 1.14 | 1.10 | 1.45 |
| 18.989 | Benzoic acid, ethyl ester (Ethyl benzoate) | 0 | 0.84 | 0 | 0 | 0 | 0 | 0 | 0 | 0 | 0 | 0 |
| 20.223 | Acetic acid, phenylmethyl ester (Benzyl acetate) | 0.00 | 0.00 | 0.00 | 0.00 | 0.00 | 0.00 | 0.00 | 0.00 | 0.00 | 0.00 | 0.00 |
| 21.035 | Methyl salicylate | 1.62 | 0.00 | 1.78 | 1.96 | 1.93 | 9.44 | 0.52 | 1.09 | 1.02 | 0.68 | 1.29 |
| 33.104 | 1,2-Benzenedicarboxylic acid, butyl octyl ester | 0.00 | 1.31 | 0.00 | 0.00 | 0.00 | 0.00 | 0.16 | 0.00 | 0.00 | 0.00 | 0.00 |
| 21.286 | Hydrazinecarboxylic acid, phenylmethyl ester | 0.96 | 0 | 0.00 | 0.00 | 0.00 | 0.00 | 0.00 | 0.00 | 0.00 | 0.00 | 0.00 |
| 14.203 | Octanoic acid, ethyl ester (Ethyl octanoate) | 0 | 0 | 0 | 0 | 0 | 0 | 0 | 0 | 0 | 0 | 0 |
| 35.247 | Dibutyl phthalate (Phthalic acid, dibutyl ester) | 0.00 | 0.25 | 0.40 | 0.00 | 0.00 | 0.49 | 0.00 | 0.00 | 0.25 | 0.00 | 0.29 |
|  | ∑ Esters | 755.66 | 4.63 | 22.25 | 819.08 | 10.63 | 305.67 | 4.97 | 2.13 | 3.34 | 7.64 | 18.60 |
| 1.674 | 2-Octanamine | 0.00 | 0.00 | 0.00 | 0.00 | 0.00 | 0.00 | 0.00 | 0.00 | 0.00 | 0.00 | 0.00 |
| 9.871 | Cyclohexene, 1-methyl-4-(1-methylethylidene)- (Terpinolene) | 0.00 | 0.00 | 0.00 | 0.00 | 0.00 | 3.48 | 0.00 | 0.00 | 0.00 | 0.25 | 0.00 |
| 14.670 | Cycloheptane | 0.00 | 0.00 | 0.00 | 0.00 | 0.00 | 0.00 | 1.14 | 0.00 | 0.00 | 0.00 | 1.41 |
| 10.095 | p-Cymene | 0.00 | 0.00 | 0.31 | 3.12 | 3.04 | 0.00 | 0.00 | 1.56 | 0.00 | 1.26 | 2.21 |
| 10.203 | o-Cymene | 0.00 | 2.17 | 1.96 | 1.08 | 1.41 | 3.27 | 0.80 | 0.67 | 0.77 | 0.63 | 0.82 |
| 10.085 | Benzene, 1-methyl-3-(1-methylethyl)- | 0.00 | 0.00 | 0.00 | 0.00 | 0.00 | 0 | 1.18 | 0.00 | 0.72 | 0.00 | 0.00 |
| 9.864 | Styrene | 0 | 0 | 0 | 0 | 2.19 | 0 | 0.00 | 0.00 | 0.00 | 0.00 | 0 |
|  | ∑ Hydrocarbons | 0.00 | 2.17 | 2.28 | 4.20 | 6.64 | 6.75 | 3.12 | 2.23 | 1.50 | 2.14 | 4.45 |
| 26.451 | p-Cresol | 0.00 | 0.00 | 0.00 | 0.00 | 0.00 | 0.51 | 0.00 | 0.00 | 0.00 | 0.00 | 0.00 |
| 28.480 | Phenol, 2-methyl-5-(1-methylethyl)- (Carvacrol) | 0.51 | 0.35 | 0.47 | 0.73 | 0.41 | 7.16 | 0.28 | 0.32 | 0.00 | 0.27 | 0.45 |
| 29.969 | 2,4-Di-tert-butylphenol | 6.26 | 4.22 | 6.11 | 6.93 | 4.55 | 6.46 | 2.28 | 3.46 | 3.91 | 3.89 | 6.08 |
| 28.057 | Thymol | 0.00 | 0.00 | 0.00 | 0.00 | 0.00 | 0 | 0.00 | 0.00 | 0.31 | 0.00 | 0.00 |
| 24.375 | Phenol, 2,6-dimethyl- | 1.37 | 0.84 | 1.12 | 2.31 | 0.96 | 0 | 0.00 | 0.85 | 0.84 | 0.66 | 0.00 |
| 25.532 | Phenol, 2-methoxy-4-propyl- (Propyl guaiacol) | 0.00 | 0.00 | 0.00 | 0.00 | 0.00 | 0 | 0.00 | 0.00 | 0.00 | 0.00 | 0.00 |
| 27.896 | Phenol, 4-ethyl- | 0.00 | 0.00 | 0.00 | 0.00 | 0.00 | 0 | 0.00 | 0.00 | 0.00 | 0.00 | 0.00 |
| 28.181 | Phenol, 2,3,5,6-tetramethyl- | 0 | 0 | 0 | 0 | 0 | 0 | 0 | 0 | 0 | 0 | 0 |
| 17.121 | 1,3-Benzenediol, 4-ethyl- (Resorcinol) | 0 | 0 | 0 | 0 | 0 | 0 | 0.30 | 0.26 | 0.14 | 0.21 | 0 |
|  | ∑ Phenols | 8.13 | 5.41 | 7.70 | 9.96 | 5.91 | 14.14 | 2.86 | 4.89 | 5.20 | 5.04 | 6.52 |
| 18.610 | 2-Toluic hydrazide | 0.00 | 0.00 | 0.00 | 0.00 | 0.00 | 0.00 | 0.00 | 0.00 | 0.00 | 0.00 | 0.00 |
| 18.607 | Phthalan | 3.44 | 4.63 | 2.90 | 0.00 | 4.22 | 14.12 | 9.39 | 2.27 | 3.27 | 2.39 | 15.49 |
| 16.145 | 2-Thiazolidinimine, 3-methyl- | 0 | 0 | 0 | 0 | 0.00 | 0.00 | 0.00 | 0.00 | 0.00 | 0.00 | 0.96 |
| 21.280 | Benzyl 2-chloroethyl sulfone | 0 | 0 | 0 | 0 | 0.00 | 0 | 0.00 | 0.00 | 0.00 | 0.00 | 0 |
|  | ∑ Miscellaneous | 3.44 | 4.63 | 2.90 | 0.00 | 4.22 | 14.12 | 9.39 | 2.27 | 3.27 | 2.39 | 16.44 |

**Table S3.** (continued).

| Rt | Compounds | S40-4 | S58-3 | S74-3 | S74-4 | Sc |
| --- | --- | --- | --- | --- | --- | --- |
| 6.235 | 1-Propanol, 2-methyl- (Isobutanol) | 1.13 | 0.00 | 14.59 | 7.74 | 28.00 |
| 8.788 | 1-Butanol, 3-methyl- (Isoamyl alcohol) | 29.25 | 2.61 | 97.15 | 93.27 | 253.34 |
| 12.342 | 1-Hexanol | 0.69 | 0.96 | 0.58 | 0.00 | 0.84 |
| 13.315 | 2-Hexanol, 2-methyl- | 0.00 | 0.00 | 1.22 | 0.00 | 7.63 |
| 15.431 | 2-Propyl-1-pentanol | 0.00 | 0.00 | 0.00 | 6.21 | 5.94 |
| 16.846 | 1-Octanol | 0.34 | 0.44 | 0.00 | 0.00 | 4.95 |
| 18.902 | 1-Undecanol | 0.00 | 0.00 | 0.00 | 0.00 | 2.93 |
| 19.526 | 9-Tetradecen-1-ol, (E)- | 0.00 | 0.00 | 0.00 | 0.00 | 0.00 |
| 22.929 | Benzyl alcohol (Phenylmethyl alcohol) | 0.25 | 0.25 | 0.00 | 0.00 | 0.00 |
| 23.516 | Phenylethyl Alcohol | 8.28 | 0.90 | 46.27 | 45.71 | 97.79 |
| 19.988 | 1-Propanol, 3-(methylthio)- | 0.00 | 0.00 | 0.48 | 0.52 | 0.00 |
| 15.424 | 1-Hexanol, 2-ethyl- | 5.62 | 6.61 | 6.01 | 0.00 | 0.00 |
| 17.685 | Terpinen-4-ol | 0.00 | 0.00 | 0.00 | 0.00 | 0.00 |
| 7.707 | Linalool | 0.00 | 0.00 | 0.00 | 0.00 | 0.00 |
| 20.840 | 1-Decanol | 0.00 | 0.00 | 0.00 | 0.00 | 2.10 |
|  | ∑ Alcohols | 45.55 | 11.77 | 166.29 | 153.45 | 403.52 |
| 14.589 | Dodecanal | 0.00 | 0.00 | 0.00 | 0.00 | 0.00 |
| 16.085 | Benzaldehyde | 0.00 | 0.00 | 0.77 | 1.05 | 0.00 |
| 1.661 | Acetaldehyde | 0.00 | 0.00 | 0.00 | 0.00 | 0.00 |
|  | ∑ Aldehydes | 0.00 | 0.00 | 0.77 | 1.05 | 0.00 |
| 10.669 | Acetoin | 0.00 | 0.00 | 2.08 | 2.59 | 0.00 |
| 16.333 | Oxacyclododecan-2-one | 0.00 | 0.00 | 0.00 | 0.00 | 0.00 |
| 17.413 | Isophorone | 2.09 | 1.45 | 1.17 | 1.34 | 1.61 |
| 25.468 | 2(3H)-Furanone, dihydro-5-pentyl- | 0.00 | 0.00 | 0.00 | 0.22 | 0.94 |
| 30.244 | Oxacyclododecan-2-one | 0.00 | 0.00 | 0.00 | 0.00 | 0.00 |
| 34.372 | 4-Acetoxy-3-methoxyacetophenone | 0.00 | 0.00 | 0.00 | 0.00 | 0.00 |
| 27.316 | 2(3H)-Furanone, 5-hexyldihydro- | 0.23 | 0.00 | 0.00 | 0.00 | 0.00 |
| 8.593 | 2-Octanone | 0.94 | 1.05 | 0.00 | 0.00 | 0.00 |
| 32.189 | Benzophenone | 0.00 | 0.00 | 0.00 | 0.00 | 0.52 |
|  | ∑ Ketones | 3.26 | 2.49 | 3.25 | 4.14 | 3.06 |
| 14.750 | Acetic acid | 0.79 | 0.64 | 2.31 | 3.90 | 4.32 |
| 17.181 | Propanoic acid, 2-methyl- (Isobutyric acid) | 0.25 | 0.00 | 0.47 | 0.54 | 2.06 |
| 19.197 | Butanoic acid, 2-methyl- (2-Methylbutyric acid) | 0.00 | 0.00 | 0.55 | 0.83 | 0.00 |
| 19.173 | Butanoic acid, 3-methyl- (Isovaleric acid) | 0.96 | 0.40 | 0.00 | 0.00 | 1.76 |
| 22.433 | 1-Hexanoic acid | 1.04 | 0.73 | 0.82 | 0.96 | 59.41 |
| 24.217 | Hexanoic acid, 2-ethyl- | 2.16 | 2.61 | 1.01 | 1.83 | 1.92 |
| 24.281 | Heptanoic acid | 0.51 | 0.14 | 0.00 | 0.00 | 0.00 |
| 26.048 | Octanoic acid | 2.32 | 0.43 | 1.21 | 1.67 | 423.16 |
| 27.732 | Nonanoic acid | 0.76 | 0.38 | 0.36 | 0.57 | 4.39 |
| 29.338 | n-Decanoic acid | 0.22 | 0.00 | 0.00 | 0.28 | 214.44 |
| 30.244 | Undecylenic acid | 0.00 | 0.00 | 0.00 | 0.00 | 22.83 |
| 31.635 | Benzoic acid | 0.20 | 0.17 | 0.33 | 0.33 | 0.00 |
| 32.360 | Dodecanoic acid | 0.00 | 0.00 | 0.00 | 0.00 | 2.85 |
| 18.379 | Butanoic acid | 0.00 | 0.00 | 0.00 | 0.00 | 0.53 |
|  | ∑ Acids | 9.20 | 5.49 | 7.07 | 10.91 | 737.66 |
| 2.379 | Ethyl Acetate | 0.00 | 0.00 | 0.94 | 1.13 | 12.62 |
| 6.440 | 1-Butanol, 3-methyl-, acetate (Isoamyl acetate) | 0.00 | 0.00 | 0.00 | 0.00 | 5.66 |
| 18.104 | Benzoic acid, methyl ester (Methyl benzoate) | 0.85 | 0.78 | 1.23 | 1.31 | 1.73 |
| 18.989 | Benzoic acid, ethyl ester (Ethyl benzoate) | 0 | 0 | 0 | 0 | 0 |
| 20.223 | Acetic acid, phenylmethyl ester (Benzyl acetate) | 0.00 | 0.00 | 0.00 | 0.00 | 0.00 |
| 21.035 | Methyl salicylate | 0.76 | 0.68 | 1.20 | 1.16 | 2.15 |
| 33.104 | 1,2-Benzenedicarboxylic acid, butyl octyl ester | 0.00 | 0.00 | 0.00 | 0.00 | 0.00 |
| 21.286 | Hydrazinecarboxylic acid, phenylmethyl ester | 0.00 | 0.00 | 0.00 | 0.00 | 0.00 |
| 14.203 | Octanoic acid, ethyl ester (Ethyl octanoate) | 0 | 0 | 0 | 0 | 91.77 |
| 35.247 | Dibutyl phthalate (Phthalic acid, dibutyl ester) | 0.00 | 0.00 | 0.00 | 0.22 | 0.87 |
|  | ∑ Esters | 1.61 | 1.46 | 3.37 | 3.82 | 114.80 |
| 1.674 | 2-Octanamine | 0.00 | 0.00 | 0.00 | 0.00 | 0.00 |
| 9.871 | Cyclohexene, 1-methyl-4-(1-methylethylidene)- (Terpinolene) | 0.00 | 0.00 | 0.00 | 0.00 | 0.00 |
| 14.670 | Cycloheptane | 0.79 | 0.00 | 0.00 | 0.00 | 3.03 |
| 10.095 | p-Cymene | 0.98 | 0.78 | 0.74 | 2.09 | 2.75 |
| 10.203 | o-Cymene | 0.51 | 0.54 | 0.97 | 0.71 | 1.00 |
| 10.085 | Benzene, 1-methyl-3-(1-methylethyl)- | 0.00 | 0.00 | 0.00 | 0.00 | 0.00 |
| 9.864 | Styrene | 0.00 | 0.00 | 0.00 | 0.00 | 0.00 |
|  | ∑ Hydrocarbons | 2.28 | 1.33 | 1.71 | 2.80 | 6.78 |
| 26.451 | p-Cresol | 0.00 | 0.00 | 0.00 | 0.00 | 0.00 |
| 28.480 | Phenol, 2-methyl-5-(1-methylethyl)- (Carvacrol) | 0.25 | 0.27 | 0.31 | 0.33 | 1.00 |
| 29.969 | 2,4-Di-tert-butylphenol | 3.40 | 3.23 | 4.59 | 5.69 | 21.03 |
| 28.057 | Thymol | 0.00 | 0.00 | 0.00 | 0.00 | 0.00 |
| 24.375 | Phenol, 2,6-dimethyl- | 0.36 | 0.34 | 1.02 | 0.80 | 0.67 |
| 25.532 | Phenol, 2-methoxy-4-propyl- (Propyl guaiacol) | 0.00 | 0.00 | 0.00 | 0.00 | 0.00 |
| 27.896 | Phenol, 4-ethyl- | 0.00 | 0.00 | 0.00 | 0.00 | 0.00 |
| 28.181 | Phenol, 2,3,5,6-tetramethyl- | 0 | 0 | 0 | 0 | 0.78 |
| 17.121 | 1,3-Benzenediol, 4-ethyl- (Resorcinol) | 0.22 | 0.33 | 0.00 | 0.00 | 0.00 |
|  | ∑ Phenols | 4.22 | 4.17 | 5.92 | 6.83 | 23.48 |
| 18.610 | 2-Toluic hydrazide | 0.00 | 0.00 | 0.00 | 0.00 | 0.00 |
| 18.607 | Phthalan | 6.14 | 4.75 | 3.22 | 4.85 | 18.92 |
| 16.145 | 2-Thiazolidinimine, 3-methyl- | 0.00 | 0.00 | 0.55 | 0.00 | 0.00 |
| 21.280 | Benzyl 2-chloroethyl sulfone | 0.00 | 0.00 | 0.00 | 0.00 | 0.00 |
|  | ∑ Miscellaneous | 6.14 | 4.75 | 3.77 | 4.85 | 18.92 |
