# Supplementary material for: Investigation of Non‐Saccharomyces Yeasts for Developing Unique Flavor Profiles in Nonalcoholic Mulberry Fermented Beverage
Source: Int J Food Sci. 2025 Sep 19;2025:5596446. doi: 10.1155/ijfo/5596446 (PMC12447106; doi:10.1155/ijfo/5596446)
Supplement: Supplementary file 4 — Supporting Information 4 Table S4: Relative odor activity values (rOAVs) of volatile aroma compounds in mulberry wines fermented with different yeast strains. [file IJFO-2025-5596446-s002.docx]

**Table S4.** Relative odor activity values (rOAVs) of volatile aroma compounds in mulberry wines fermented with different yeast strains.

|  | Isobutanol | Isoamyl alcohol | 1-Hexanol | 1-Octanol | Benzyl alcohol | Phenylethyl Alcohol | 1-Hexanol, 2-ethyl- | Terpinen-4-ol | Linalool | 1-Decanol | Acetic acid | Isobutyric acid | Isovaleric acid |
| --- | --- | --- | --- | --- | --- | --- | --- | --- | --- | --- | --- | --- | --- |
| CAS | 78-83-1 | 123-51-3 | 111-27-3 | 111-87-5 | 100-51-6 | 60-12-8 | 104-76-7 | 562-74-3 | 78-70-6 | 112-30-1 | 64-19-7 | 79-31-2 | 503-74-2 |
| Odor threshold (μg/L) | 40000 ^1^ | 30000 ^2^ | 500 ^3^ | 110 ^3^ | 620 ^4^ | 1100 ^3^ | 270 ^3^ | 100 ^2^ | 15 ^2^ | 400 ^5^ | 200000 ^1^ | 2300 ^1^ | 33.4 ^1^ |
| Category | C_4_H_10_O | C_5_H_12_O | C_6_H_14_O | C_8_H_18_O | C_7_H_8_O | C_8_H_10_O | C_8_H_18_O | C_10_H_18_O | C_10_H_18_O | C_10_H_22_O | C_2_H_4_O_2_ | C_4_H_8_O_2_ | C_5_H_10_O_2_ |
| S64-2 | 0.00 | 0.01 | 0.00 | 0.10 | 0.06 | 0.24 | 0.00 | 0.00 | 0.00 | 0.00 | 0.00 | 0.00 | 0.00 |
| S11-4 | 0.00 | 0.41 | 0.00 | 0.66 | 0.00 | 7.54 | 1.96 | 0.00 | 0.00 | 0.00 | 0.00 | 0.16 | 36..85 |
| S11-5 | 0.06 | 0.47 | 0.00 | 0.66 | 0.00 | 7.89 | 2.13 | 0.00 | 0.00 | 0.00 | 0.00 | 0.17 | 40.08 |
| S14-5(2) | 0.03 | 0.24 | 0.00 | 0.43 | 0.00 | 4.35 | 1.09 | 0.00 | 0.00 | 0.00 | 0.00 | 0.09 | 18.62 |
| S62-3 | 0.04 | 0.30 | 0.00 | 0.37 | 0.00 | 4.81 | 1.14 | 0.00 | 0.00 | 0.00 | 0.00 | 0.09 | 17.39 |
| S64-3 | 0.01 | 0.15 | 0.07 | 0.43 | 0.00 | 1.94 | 1.07 | 0.00 | 0.00 | 0.00 | 0.00 | 0.01 | 0.00 |
| S76-1 | 0.04 | 0.28 | 0.00 | 0.37 | 0.00 | 4.53 | 1.12 | 0.00 | 0.00 | 0.00 | 0.00 | 0.09 | 20.47 |
| S76-3 | 0.04 | 0.29 | 0.00 | 0.44 | 0.00 | 5.21 | 1.14 | 0.00 | 0.00 | 0.00 | 0.00 | 0.12 | 18.32 |
| G27-1 | 0.07 | 0.51 | 0.00 | 0.00 | 0.00 | 10.70 | 6.99 | 0.00 | 0.00 | 0.00 | 0.00 | 0.34 | 46.72 |
| Sc | 0.00 | 0.05 | 0.01 | 0.25 | 0.00 | 0.48 | 0.00 | 0.00 | 0.00 | 0.03 | 0.00 | 0.00 | 0.29 |

Note:

| ^1^ Francis and Newton (2005) |
| --- |
| ^2^ Slaghenaufi et al. (2021) |
| ^3^ Wei et al. (2018) |
| ^4^ Blanco et al. (2020) |
| ^5^ Zhang et al. (2013) |
| ^6^ Avellone et al. (2018) |

**Table S4.** (continued).

|  | 1-Hexanoic acid | Octanoic acid | Nonanoic acid | n-Decanoic acid | Dodecanoic acid | Butanoic acid | Ethyl Acetate | Isoamyl acetate | Methyl benzoate | Ethyl benzoate | Methyl salicylate | Ethyl octanoate |
| --- | --- | --- | --- | --- | --- | --- | --- | --- | --- | --- | --- | --- |
| CAS | 142-62-1 | 124-07-2 | 112-05-0 | 334-48-5 | 143-07-7 | 107-92-6 | 141-78-6 | 123-92-2 | 93-58-3 | 93-89-0 | 119-36-8 | 106-32-1 |
| Odor threshold (μg/L) | 420 2 | 500 2 | 3000 6 | 1000 1 | 1000 4 | 240 6 | 7500 4 | 30 2 | 0.52 3 | 60 3 | 50 2 | 5 2 |
| Category | C_6_H_12_O_2_ | C_8_H_16_O_2_ | C_9_H_18_O_2_ | C_10_H_20_O_2_ | C_12_H_24_O_2_ | C_4_H_8_O_2_ | C_4_H_8_O_2_ | C_7_H_14_O_2_ | C_8_H_8_O_2_ | C_9_H_10_O_2_ | C_8_H_8_O_3_ | C_10_H_20_O_2_ |
| S64-2 | 0.01 | 0.15 | 0.01 | 0.06 | 0.00 | 0.00 | 0.23 | 100.00 | 0.00 | 0.00 | 1.05 | 0.00 |
| S11-4 | 0.37 | 2.24 | 0.07 | 0.14 | 0.00 | 0.00 | 5.52 | 100.00 | 97.32 | 0.00 | 2.04 | 0.00 |
| S11-5 | 0.38 | 2.28 | 0.07 | 0.14 | 0.00 | 0.00 | 5.74 | 79.74 | 100.00 | 0.00 | 1.99 | 0.00 |
| S14-5(2) | 0.19 | 1.25 | 0.04 | 0.07 | 0.00 | 0.00 | 3.24 | 33.34 | 100.00 | 0.00 | 1.13 | 0.00 |
| S62-3 | 0.23 | 1.71 | 0.04 | 0.10 | 0.00 | 0.00 | 3.63 | 35.50 | 100.00 | 0.00 | 1.24 | 0.00 |
| S64-3 | 0.03 | 0.13 | 0.02 | 0.02 | 0.00 | 0.00 | 3.02 | 27.94 | 100.00 | 0.00 | 2.35 | 0.00 |
| S76-1 | 0.23 | 1.48 | 0.04 | 0.09 | 0.00 | 0.00 | 3.59 | 37.42 | 100.00 | 0.00 | 1.20 | 0.00 |
| S76-3 | 0.13 | 0.94 | 0.04 | 0.09 | 0.00 | 0.00 | 4.02 | 29.74 | 100.00 | 0.00 | 1.33 | 0.00 |
| G27-1 | 0.00 | 8.86 | 0.23 | 0.65 | 0.00 | 0.00 | 19.41 | 100.00 | 0.00 | 0.00 | 7.12 | 0.00 |
| Sc | 0.77 | 4.61 | 0.01 | 1.17 | 0.02 | 0.01 | 0.01 | 1.03 | 18.17 | 0.00 | 0.23 | 100.00 |
